# The effect of milk type and fortification on the growth of low‐birthweight infants: An umbrella review of systematic reviews and meta‐analyses

**DOI:** 10.1111/mcn.13176

**Published:** 2021-03-17

**Authors:** Krysten North, Megan Marx Delaney, Carl Bose, Anne C. C. Lee, Linda Vesel, Linda Adair, Katherine Semrau

**Affiliations:** ^1^ Department of Pediatrics University of North Carolina at Chapel Hill Chapel Hill North Carolina USA; ^2^ Ariadne Labs Brigham Women's Hospital and Harvard TH Chan School of Public Health Boston Massachusetts USA; ^3^ Department of Pediatric Newborn Medicine Brigham and Women's Hospital Boston Massachusetts USA; ^4^ Harvard Medical School Boston Massachusetts USA; ^5^ Department of Nutrition, Carolina Population Center, Gillings School of Public Health University of North Carolina at Chapel Hill Chapel Hill North Carolina USA

**Keywords:** formula, fortification, growth, human milk, infant, low birthweight, umbrella review

## Abstract

Approximately 15% of infants worldwide are born with low birthweight (<2500 g). These children are at risk for growth failure. The aim of this umbrella review is to assess the relationship between infant milk type, fortification and growth in low‐birthweight infants, with particular focus on low‐ and lower middle–income countries. We conducted a systematic review in PubMed, CINAHL, Embase and Web of Science comparing infant milk options and growth, grading the strength of evidence based on standard umbrella review criteria. Twenty‐six systematic reviews qualified for inclusion. They predominantly focused on infants with very low birthweight (<1500 g) in high‐income countries. We found the strongest evidence for (1) the addition of energy and protein fortification to human milk (donor or mother's milk) leading to increased weight gain (mean difference [MD] 1.81 g/kg/day; 95% confidence interval [CI] 1.23, 2.40), linear growth (MD 0.18 cm/week; 95% CI 0.10, 0.26) and head growth (MD 0.08 cm/week; 95% CI 0.04, 0.12) and (2) formula compared with donor human milk leading to increased weight gain (MD 2.51 g/kg/day; 95% CI 1.93, 3.08), linear growth (MD 1.21 mm/week; 95% CI 0.77, 1.65) and head growth (MD 0.85 mm/week; 95% CI 0.47, 1.23). We also found evidence of improved growth when protein is added to both human milk and formula. Fat supplementation did not seem to affect growth. More research is needed for infants with birthweight 1500–2500 g in low‐ and lower middle–income countries.

**Key messages**

- Energy (fat or carbohydrate) and protein fortification of human milk is associated with increased growth in low‐birthweight infants during birth hospitalization, although not associated with increased growth between discharge and 6 months.
- Formula compared with donor human milk is associated with increased growth in low‐birthweight infants.
- Most low birthweight feeding studies have focused on infants with a birthweight <1500 g; only a few focus on infants with birthweight 1500 to <2500 g, a group with a unique nutritional profile.
- Only a small percentage of the studies of nutritional interventions for low‐birthweight infants have been conducted in low‐ and lower middle–income countries.

## 1 INTRODUCTION

Approximately 15% of infants are born with low birthweight (LBW) (<2500 g) (Blencowe et al., 2019). Compared with normal‐birthweight infants, LBW infants have increased risk for morbidity and mortality. Small size at birth contributes to 80% of neonatal deaths (Lawn et al., 2014), and LBW infants frequently experience poor postnatal growth (Cooke et al., 2004). Early growth failure has been associated with poor outcomes, including negative effects on neurodevelopment (Ehrenkranz et al., 2006). Optimal nutrition for LBW infants, both during their initial hospitalization after birth and after their discharge to home, is important for survival, growth and normal development.

In 2011, the World Health Organization (WHO) published a broad review of LBW nutrition, *Guidelines on Optimal Feeding for Low‐Birthweight Infants in Low‐ and Middle‐Income Countries*. The guidelines recommended mother's unfortified milk as the initial option for feeding LBW infants, with donor human milk being the next best choice if mother's milk is not available. Fortification of human milk was recommended only in the case of inadequate weight gain. Notably, most of the studies included in this WHO review were judged to be of poor quality, such that 13 of the 18 guidelines (72%) are based on ‘weak’ or ‘weak situational’ evidence.

A number of systematic reviews of the feeding of LBW infants have been published since the establishment of the WHO guidelines. We chose to conduct an umbrella review, an overview of systematic reviews, to coalesce the data on a large number of feeding interventions. Umbrella reviews are used to synthesize evidence on a broad topic and facilitate decision making (Biondi‐Zoccai, 2016). The objective of this umbrella review is to summarize the available review literature on the relationship between milk options for LBW infants, including human milk, infant formula and infant milk fortifiers, and growth up to 6 months post‐term. We hope that this evidence synthesis may provide guidance for the formation of feeding recommendations, while acknowledging that many other factors, such as morbidities including necrotizing enterocolitis, cost and feasibility, are also important considerations to guide feeding choices.

The prevalence of LBW is disproportionately high in low‐ and middle‐income countries (LMICs). An estimated 91% of LBW infants are born in LMICs (Blencowe et al., 2019). Given the size and vulnerability of this population, we were particularly interested in principles for feeding LBW infants that are tailored to resource‐limited environments. In undertaking this umbrella review, we anticipated that the bulk of research on the feeding of LBW infants has been conducted in high‐income settings. Because we were interested in conducting a comprehensive search, we chose not to restrict our inquiry to LMICs but to pay particular attention to the results stemming from this group. Because the preponderance of LBW infants surviving from LMICs fall into the 1500–2500 g weight band, we have a special interest in infants with these birthweights. As much as possible, we wanted to synthesize the available evidence to formulate recommendations for feeding LBW infants in LMICs, while acknowledging the limitations in extrapolating principles between populations.

## 2 METHODS

We followed the Preferred Reporting Items for Systematic Reviews and Meta‐Analyses (PRISMA) guidelines while conducting this review (see Data S1). We registered the protocol for this review with PROSPERO prior to review initiation and submitted updates for protocol modifications. The full protocol is available in the Data S1.

### 2.1 Search strategy

We conducted a search of Medline, CINAHL, Embase and Web of Science databases. The initial query was done in Medline, CINAHL and Embase in December 2018 with the addition of studies from the Web of Science in March 2019. The search was updated in January 2020. Search terms included probes for ‘low birthweight’, ‘premature’, ‘small for gestational age’, ‘breast milk’, ‘infant formula’, and ‘systematic review’, in addition to a number of related terms (see Data S1). We limited our selection to articles published in English and only included systematic reviews or meta‐analyses. We had no limitations on publication dates. If multiple versions of a systematic review were available, we only included the most recently updated version. We only included reviews for which full‐text articles were available and did not include studies from the grey literature.

### 2.2 Population, intervention and outcomes

Our population of interest was LBW infants. As such, we limited our umbrella review population to reviews primarily targeted to preterm infants or those with birthweight <2500 g. We calculated a pooled weighted average birthweight among the primary studies included in each review both to see if the population met the inclusion criteria (<2500 g) and to better understand the profile of the population represented by the review.

We considered interventions relating to infant milk options, including types of milk such as formula or human milk and milk fortification with macronutrients including added fat, carbohydrate or protein components. Reviews only addressing vitamin or mineral fortification were excluded. We included both inpatient and outpatient interventions. Comparison groups varied by review, but all included unfortified mother's own milk and/or other infant milk and fortification options.

Our outcome of interest was growth assessed through measurement of weight, length, head circumference, body composition, skinfold thickness or fat‐free mass. Reviews that did not report anthropometrics were excluded. We included growth outcomes from birth up to 6 months post‐term.

### 2.3 Study selection and data extraction

Two reviewers (K. N. and M. M. D.) independently screened eligible articles first by title and abstract, then full text. We determined inclusion based on population, intervention and outcome criteria discussed previously. Any screening conflicts were resolved through discussion between the two reviewers and adjudication by a third reviewer (K. E. A. S.). In accordance with PRISMA guidelines, two reviewers (K. N. and M. M. D.) independently completed data extraction for 50% of the included studies and independently achieved >80% agreement (K.N. and M. M. D.), with a single reviewer extracting data from the remainder of the studies (K. N.). See the Data S1 for a complete list of extracted information.

### 2.4 Data analysis

We evaluated the risk of bias of selected reviews using the ‘A Measurement Tool to Assess Systematic Reviews (AMSTAR 2)’ checklist, a quality assessment tool specifically designed for systematic reviews (Shea et al., 2017). The strength of evidence from every unique meta‐analysis was graded on the basis of conventions established in other umbrella reviews (Belbasis et al., 2015; Bellou et al., 2017; Fusar‐Poli & Radua, 2018). We extracted all data used to grade the evidence from the reviews. We did not calculate a pooled effect size between reviews. The evidence was classified as follows:

- Convincing: fixed‐ or random‐effects *P*‐value <0.00001, population size >500, 95% confidence interval excludes null, heterogeneity *I* ^2^ value <50%.
- Highly suggestive: does not meet criteria for convincing and fixed‐ or random‐effects *P*‐value <0.00001, population size >500, largest study excludes null.
- Suggestive: does not meet criteria for convincing and fixed‐ or random‐effects *P*‐value <0.001, population size >500.
- Weak: does not meet criteria for convincing and fixed‐ or random‐effects *P*‐value <0.05
- Not significant: fixed‐ or random‐effects *P*‐value >0.05

## 3 RESULTS

**FIGURE 1:**
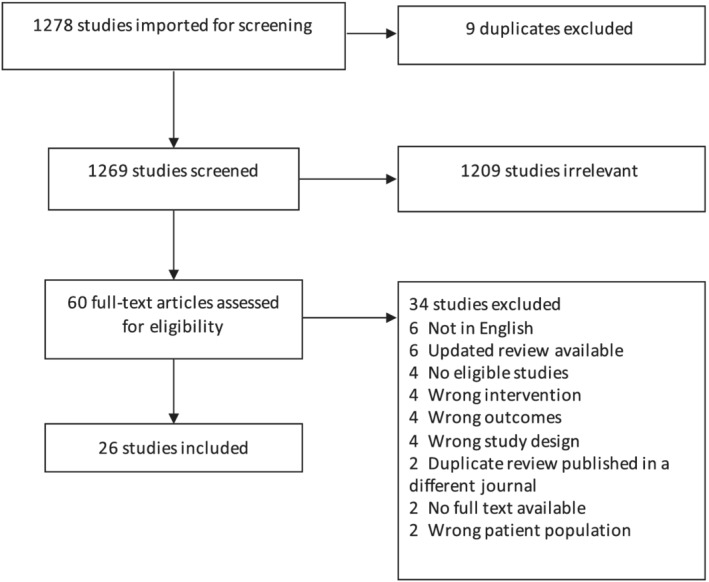
Flow chart of selection of eligible reviews

We screened the titles and abstracts of 1278 references. Sixty full‐text articles were reviewed, and 26 reviews met eligibility criteria for data extraction (Figure 1). A list of full text articles that were not included as well as reasons for elimination is shown in the Data S1. These 26 review articles included 150 unique studies. Some of these studies were included in more than one systematic review. A list of individual studies included in multiple reviews is shown in the Data S1.

### 3.1 Characteristics of included reviews

The included reviews address a number of feeding options for LBW infants (Table 1). Twenty‐one of the 26 reviews had a pooled weighted average birthweight of 900–1499 g. Four reviews had a pooled weighted average birthweight between 1500 and <2500 g. Twenty‐three reviews limited their population to studies of premature infants. One review specifically addressed infants who were term but small for gestational age (SGA) (Santiago et al., 2019). Twenty reviews reported the country or region that was the setting of the primary studies. Of these, 98% (147/150) of primary studies were conducted in upper middle– or high‐income countries or predominantly high‐income regions. Two per cent of primary studies (3/150) occurred in India, a lower middle–income country. No studies occurred in low‐income countries. See the Data S1 for additional study details. Nineteen reviews conducted formal meta‐analyses, whereas seven reviews presented outcomes as a narrative summary of included studies. Some reviews included population‐based or non‐randomized studies (Data S1). Authors were limited in the conclusions that could be drawn from these studies. Studies without a control population were not included in meta‐analyses and thus did not impact the effect size.

**TABLE 1:** Characteristics of the 26 included reviews

Author (year)	Population	Intervention	Comparison group	AMSTAR Rating
Donor human milk				
Boyd (2007)	Preterm or LBW infants Setting: United Kingdom (UK) × 2, Finland, Hungary, United Stated of America (USA), France	Donor human milk including (1) Sole diet of donor milk (2) Mother's own milk supplemented with donor milk (3) Fortified donor milk	Infant formula including (1) Sole diet of formula (2) Mother's own milk supplemented with formula (3) Fortified formula	Moderate quality
Quigley (2019)	Preterm or LBW infants Setting: Italy, USA × 4, Austria, UK × 3, Canada, Finland, Hungary	Donor human milk including (1) Sole diet of donor milk (2) Mother's own milk supplemented with donor milk	Infant formula including (1) Sole diet of formula (2) Mother's own milk supplemented with formula	Moderate quality
Yu (2019)	Infants with birthweight <1500 g Setting: USA × 3, France	Donor human milk	Infant formula	Moderate quality
Exclusive breastfeeding				
Santiago (2019)	Term infants who are small for gestational age Setting: England × 3, India, Spain	Exclusive breastfeeding	Breastfeeding alternatives including fortified breast milk, term formula and preterm formula	Low quality
Multinutrient supplementation				
Brown (2016)	Preterm or LBW infants Setting: Oman, Denmark, India × 2, USA × 3, UK × 2, South Africa × 2, Sweden, Italy, Canada	Human milk (mother's own or donor) fortified with both energy (carbohydrate or fat) and protein	Human milk without energy or protein fortification	Moderate quality
Walsh (2019)	Preterm infants Setting: USA × 2, UK × 2, Thailand, South Africa, Turkey	Formula fortified with both energy (>72 kcal/100 ml) and protein (>1.7 g/100 ml)	Standard formula with energy ≤ 72 kcal/100 ml and protein content ≤ 1.7 g/100 ml	Moderate quality
Young (2013)	Preterm or LBW infants after hospital discharge Setting: Not reported	Human milk fortified with more than one nutrient: protein, fat, carbohydrate, or minerals	Unfortified human milk	Moderate quality
Young (2016)	Preterm infants after hospital discharge Setting: Italy × 3, North America, UK × 3, Taiwan, France, Belgium, South Korea, USA, Israel, Not reported × 3	Multinutrient‐enriched infant formula including(1) Postdischarge formula fortified with energy (72–74 kcal/100 ml) and protein (1.8–1.9 g/100 ml)(2) Preterm formula fortified with energy (~80 kcal/100 ml) and protein (2.0–2.4 g/100 ml)	Standard term formula with energy 66–68 kcal/100 ml and protein 1.4–1.7 g/100 ml	Moderate quality
Teller (2016)	Preterm infants after hospital discharge Setting: Not reported	Preterm formula (~80 kcal/100 ml) or nutrient enriched standard term formula (60–70 kcal/100 ml but added protein) or postdischarge formula (70–79 kcal/100 ml)	Term formula with energy 60–70 kcal/100 ml	Low quality
Carbohydrate supplementation				
Amissah (2018)	Preterm infants Setting: Iran	Human milk with carbohydrate fortification (non‐human short‐chain galato‐oligosaccharides/long‐chain fructo‐oligosaccharides supplement)	Unfortified human milk	Moderate quality
Fat supplementation				
Amissah (2018)	Preterm infants Setting: Sweden	Human milk with fat fortification (1 g human milk fat/100 ml)	Unfortified human milk	Moderate quality
Gibson (2001)	Preterm infants Setting: Not reported	Infant formula fortified with long chain polyunsaturated fatty acids (LC PUFA)	Infant formula without LC PUFA	Low quality
Moon (2016)	Preterm infants Setting: USA × 3, Netherlands, Canada, Taiwan, UK × 2, Not specified × 7	Infant formula fortified with LC PUFA	Infant formula without LC PUFA	Low quality
Nehra (2002)	Preterm, appropriate‐for‐gestational‐age sized infants Setting: Not reported	High MCT formula (exclusive diet)	Low MCT formula (exclusive diet)	Critically low quality
Newberry (2016)	Preterm infants Setting: Canada, Australia × 2, USA, Norway, Netherlands	Infant formula fortified with omega‐3 fatty acids	Infant formula without omega‐3 fatty acid fortification	Moderate quality
Rodriguez (2012)	Preterm infants Setting: Not reported	Infant formula fortified with omega‐3 fatty acids	Infant formula fortified with omega‐3 fatty acids	Low quality
Udell (2005)	Preterm infants Setting: USA × 2, France	Infant formula with alpha‐linolenic acid and linolenic fatty acid fortification	Infant formula without alpha‐linolenic acid and linolenic acid fatty acid fortification	Critically low quality
Protein supplementation				
Amissah (2018)	Preterm infants Setting: Europe × 3, USA, Not specified × 2	Human milk with protein fortification	Unfortified human milk	Moderate quality
Cao (2018)	LBW infants Setting: USA × 4, Germany × 2, Italy, Netherlands, Canada	Infant formula fortified with taurine	Infant formula without taurine	Low quality
Fenton (2014)	LBW infants Setting: Not reported	Formula composed of varying protein concentrations including: (1) Low protein intake (<3.0 g/kg/day) (2) High protein intake of equal to of greater than 3.0 g/kg/day but less than 4.0 g/kg/day (3) Very high protein intake of equal to or greater than 4.0 g/kg/day	Intergroup comparison	Moderate quality
Liu (2015)	Infants with birth weight ≤1750 g, gestational age ≤34 weeks Setting: Australia, USA × 2, Macedonia, Turkey	Human milk fortified with human milk fortifier (HMF) containing higher‐than‐standard protein content.	Human milk fortified with standard HMF	Low quality
Moe‐Byrne (2016)	Preterm infants Setting: USA × 2, Turkey, Netherlands	Infant milk fortified with glutamine	Infant milk not fortified with glutamine	Moderate quality
Pimpin (2019)	Preterm infants Setting: Canada, Netherlands, USA × 2, UK × 2	Animal protein‐fortified infant formula or human milk	Infant formula with lower protein content or unfortified human milk	Moderate quality
Tonkin (2014)	Preterm infants who are also LBW Setting: Spain, UK × 2, Netherlands, USA × 9, Ireland, Canada × 2, Sweden × 2, Finland, Australia, Italy × 2, Not specified × 2	Infant milk with higher protein content including (1) Formula with high protein (2) Protein‐fortified human milk	Infant milk with lower protein content including (1) Formula with lower protein (2) Unfortified human milk (3) Fortified human milk with lower protein HMF	Moderate quality
Hydrolyzation				
Ng (2019)	Preterm infants Setting: Germany × 3, France, Italy, USA	Hydrolysed cow's milk formula	Non‐hydrolysed cow's milk formula	Moderate quality
Tan‐Dy (2013)	Preterm infants Setting: Canada	Infant milk with lactase	Infant milk with placebo or no intervention	Moderate quality

Abbreviations: AMSTAR 2, A Measurement Tool to Assess Systematic Reviews version 2; HMF, human milk fortifier; LBW, low birthweight; LC PUFA, long‐chain polyunsaturated fatty acids; MCT, medium‐chain triglycerides.

### 3.2 Quality assessment of included reviews

**FIGURE 2:**
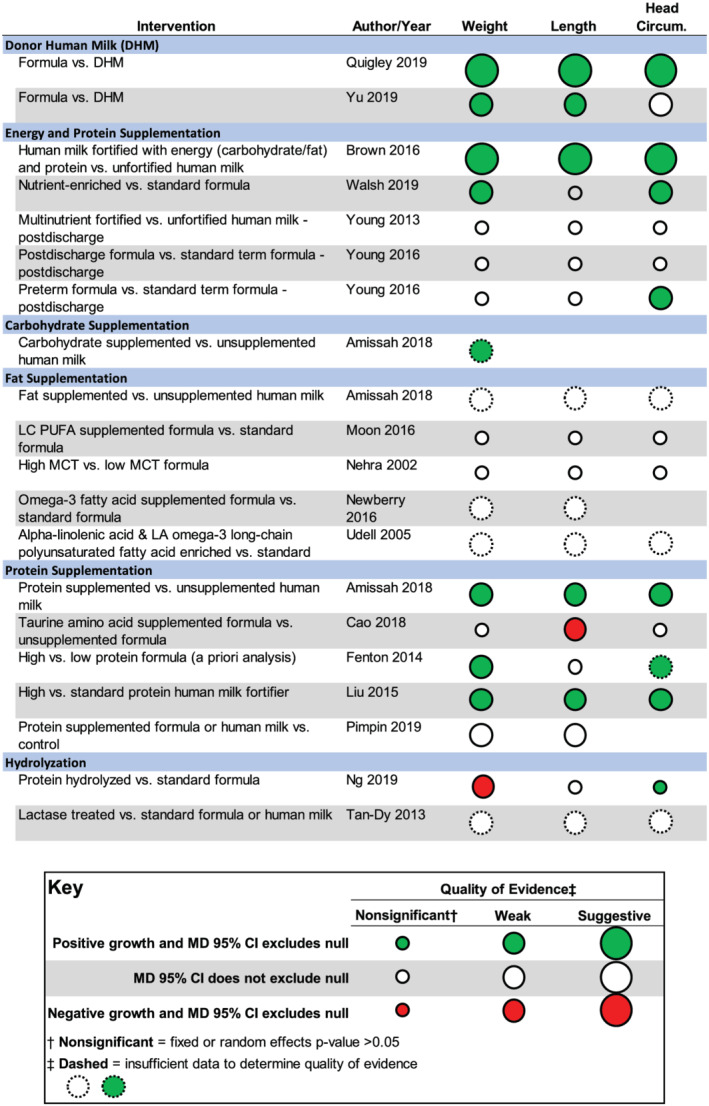
Outcomes and quality of evidence for key meta‐analyses included in this umbrella review are grouped by type of intervention. The direction and significance of the weighted mean difference are indicated by the colour of the circle. The quality of the evidence is indicated by the size of the circle. See key for further detail. Abbreviations: CI, confidence interval; Circum, circumference; DHM, donor human milk; LA, linoleic acid; LC PUFA, long‐chain polyunsaturated fatty acids; MCT, medium‐chain triglycerides; MD, mean difference

None of the included reviews met AMSTAR 2 criteria to be considered high quality. Seventeen of the 26 systematic reviews were of moderate quality. Seven reviews were considered low quality, and two were considered critically low quality. See Figure 2 and Table 1 for AMSTAR 2 results.

### 3.3 Outcomes by intervention

**FIGURE 3:**
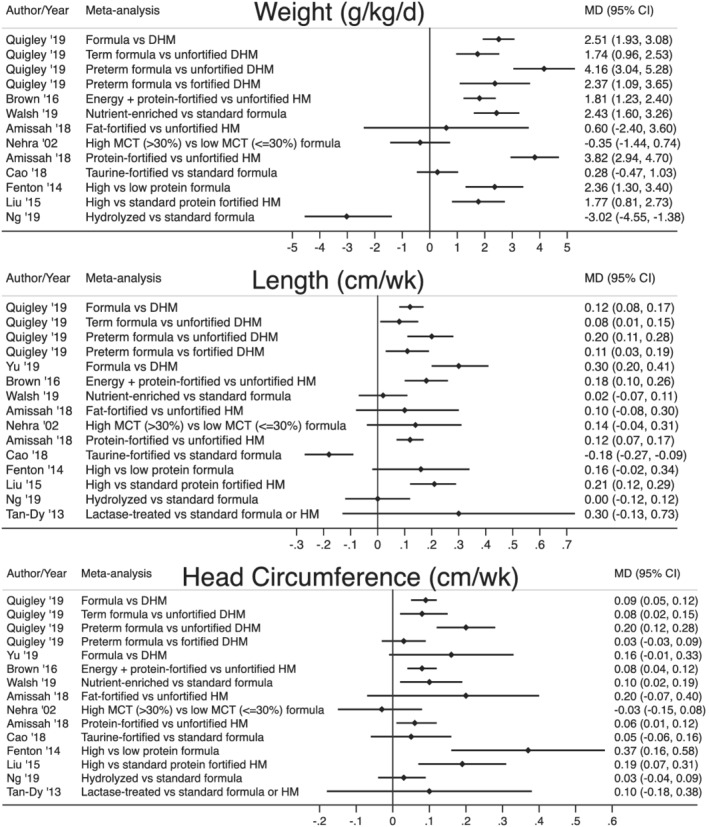
Effect size and 95% confidence intervals of key meta‐analyses demonstrating the mean difference in weight gain, linear growth and head growth between feeding groups. Abbreviations: MD, mean difference; CI, confidence interval; g/kg/d, grams/kilogram/day; cm/wk, centimetres/week; DHM, donor human milk; HM, human milk; MCT, medium chain triglyceride

Figure 2 summarizes key outcomes including the quality of evidence. The effect size and 95% confidence intervals of key meta‐analyses are depicted as forest plots in Figure 3. See Data S1 for detailed growth outcomes of individual reviews.

#### 3.3.1 Donor human milk compared with formula

Three reviews comparing donor human milk with formula found consistently greater growth in the formula group (Boyd et al., 2007;Quigley et al., 2018 ; Yu et al., 2019). In a systematic review by Quigley et al., the formula group demonstrated greater weight gain during birth hospitalization in the neonatal unit compared with the donor human milk group (mean difference [MD] 2.51 g/kg/day; 95% confidence interval [CI] 1.93, 3.08), linear growth (MD 1.21 mm/week; 95% CI 0.77, 1.65), and head growth (MD 0.85 mm/week; 95% CI 0.47, 1.23) (Quigley et al., 2018; Yu et al., 2019). In a subgroup analysis comparing formula versus donor human milk as a supplement to mother's own milk, weight gain significantly improved with formula (MD 2.22 g/kg/day; 95% CI 1.23, 3.21), but linear growth (MD 0.67 mm/week; 95% CI −0.04, 1.38) and head growth (MD 0.24 mm/week; 95% CI −0.32, 0.80) were no longer significantly different for formula compared with donor human milk (Quigley et al., 2018). A review by Yu et al. also showed improved growth in the formula versus donor human milk group for weight gain (MD 6.58 g/day; 95% CI 1.98, 11.19) and linear growth (MD 0.30 cm/week; 95% CI 0.20, 0.41). In a subgroup analysis of growth by birthweight category, both the group of infants with birthweight <1000 g and infants with birthweight 1000–1500 g demonstrated significantly greater weight gain in the formula group (birthweight <1000 g: MD 2.80 g/day; 95% CI 1.20, 4.39; birthweight 1000–1500 g: MD 10.42 g/day; 95% CI 8.53, 12.30) (Yu et al., 2019). Boyd and colleagues did not perform a meta‐analysis but presented a narrative summary of studies comparing donor human milk and formula. Most studies in this review found greater weight, length, head circumference and skinfold thickness gains in the formula group when given as either an exclusive diet or as a supplement to mother's own milk (Boyd et al., 2007).

#### 3.3.2 Exclusive breastfeeding

A single review compared exclusive breastfeeding with a number of other infant feeding options for full‐term infants who were small for gestational age (Santiago et al., 2019). Santiago et al. did not conduct a meta‐analysis, but the individual studies in this review demonstrated heterogeneous findings with regard to weight gain and linear growth in infants fed human milk, fortified human milk, preterm formula and term formula (Santiago et al., 2019).

#### 3.3.3 Hydrolyzed infant milk

Two reviews investigated the impact of hydrolyzed feeds on infant growth. Hydrolyzed protein formula, in which the milk proteins have been chemically or enzymatically digested to oligopeptides, was associated with significantly lower weight gain (MD −3.02 g/kg/day; 95% CI −4.55, −1.38) compared with standard formula, although linear growth (MD −0.04 mm/week; 95% CI −1.24, 1.15) and head growth (MD 0.27 mm/week; 95% CI −0.39, 0.94) were not different between the groups (Ng et al., 2019). Infant formula and human milk treated with lactase compared with standard formula and human milk demonstrated no significant difference in weight gain (day 14: MD 2.7 g/day; 95% CI −1.47, 6.87; study exit: MD 2.2 g/day; 95% CI −0.98, 5.3), linear growth (day 14 or study exit: MD 0.30 cm/week; 95% CI −0.13, 0.73) or head circumference (day 14 or study exit: MD 0.10 cm/week; 95% CI −0.18, 0.38) (Tan‐Dy & Ohlsson, 2013).

#### 3.3.4 Energy and protein fortification of infant milk

Several reviews investigated the impact of energy (fat or carbohydrate) and protein fortification of either human milk or formula and found significantly higher growth during birth hospitalization. Brown et al. examined energy and protein fortification of human milk during the birth hospitalization of preterm infants. They found greater weight gain (MD 1.81 g/kg/day; 95% CI 1.23, 2.40), linear growth (MD 0.18 cm/week; 95% CI 0.10, 0.26) and head growth (MD 0.08 cm/week; 95% CI 0.04, 0.12) in the fortified group (Brown et al., 2016; Walsh et al., 2019). Walsh et al. examined the effect of energy/protein fortification of formula during birth hospitalization on preterm infant growth. They found greater weight gain (MD 2.43 g/kg/day; 95% CI 1.60, 3.26) and head growth (MD 1.04 mm/week; 95% CI 0.18, 1.89) but no difference in linear growth (MD 0.22 mm/week; 95% CI −0.70, 1.13) in the fortified group.

However, energy and protein fortification seemed to have little impact on preterm infants after they left the hospital. Three reviews specifically investigated the impact of energy and protein fortification on postdischarge growth (Teller et al., 2016; Young et al., 2013; Young et al., 2016). Infants fed either postdischarge formula (about 72–74 kcal/100 ml) or preterm formula (about 80 kcal/100 ml) compared with standard formula (about 66–68 kcal/100 ml) after discharge from the hospital did not have greater 6‐month weight (postdischarge formula: MD 35.54 g; 95% CI −113.71, 184.78; preterm formula: MD 74.60 g; 95% CI −164.73, 313.92), length (postdischarge formula: MD 2.12 mm; 95% CI −2.16, 6.41; preterm formula: MD 1.83 mm; 95% CI −6.25, 9.92), or head circumference (postdischarge formula: MD 2.28 mm; 95% CI −0.28, 4.83) except for higher head circumference in the preterm formula group (MD 5.82 mm; 95% CI 1.32, 10.32) (Young et al., 2016). Similarly, a qualitative systematic review found mixed results of energy and protein‐fortified formula on growth (Teller et al., 2016). Meta‐analysis of energy and protein‐fortified human milk also found no impact on postdischarge weight (MD 138.26 g; 95% CI −89.87, 366.40), length (MD 0.06 cm; 95% CI −0.14, 1.33), or head circumference at 3 to 4 months post‐term (MD 0.22 cm; 95% CI −0.15, 0.58) (Young et al., 2013).

#### 3.3.5 Carbohydrate only fortification

One review investigated carbohydrate fortification of human milk with a nonhuman short‐chain galacto‐oligosaccharide/long‐chain fructo‐oligosaccharide supplement (Amissah et al., 2018a). The weight in the intervention group was higher at 30 days compared with infants fed nonfortified human milk (MD 160.4 g; 95% CI 12.4, 308.4). Other growth metrics were not reported.

#### 3.3.6 Fat only fortification

Several reviews analysing fat fortification of both human milk and formula found that it makes no difference in growth. Five reviews specifically addressed the effect of long‐chain polyunsaturated fatty acids (LC PUFA) (Gibson et al., 2001; Moon et al., 2016; Newberry et al., 2016; Rodríguez et al., 2012; Udell et al., 2005). Three meta‐analyses of formula fortified with LC PUFA showed no statistically significant effect on weight, length or head circumference within the first 6 months of life (Moon et al., 2016; Newberry et al., 2016; Udell et al., 2005). Two systematic reviews described mixed results in a qualitative assessment (Gibson et al., 2001; Rodríguez et al., 2012). A meta‐analysis of general fat fortification found no impact on growth, though only one small study was reported (weight gain: MD 0.60 g/kg/day; 95% CI −2.4, 3.6; linear growth: MD 0.1 cm/week; 95% CI −0.08, 0.3; head growth: MD 0.2 cm/week; 95% CI −0.07, 0.40) (Amissah et al., 2018b). A review of high versus low medium‐chain triglyceride fortification found no difference in weight gain (MD −0.35 g/kg/day; 95% CI −1.44, 0.74), linear growth (MD 0.14 cm/week; 95% CI −0.04, 0.31), head growth (MD −0.03 cm/week; 95% CI −0.15, 0.08), or increase in skinfold thickness (MD −0.15 mm/week; 95% CI −0.41, 0.11) (Nehra et al., 2002).

#### 3.3.7 Protein and amino acid fortification

Five reviews examined the impact of protein fortification on growth; most reported increased growth associated with protein supplementation (Amissah et al., 2018c; Fenton et al., 2014; Liu et al., 2015; Pimpin et al., 2019; Tonkin et al., 2014). A review of protein‐fortified versus unfortified human milk reported increased growth in the intervention group (weight gain: MD 3.82 g/kg/day; 95% CI 2.94, 4.70; linear growth: MD 0.12 cm/week; 95% CI 0.07, 0.17; head growth: MD 0.06 cm/week; 95% CI 0.01, 0.12) (Amissah et al., 2018c). Similarly, a review of high‐ versus low‐protein fortification of human milk found increased weight gain, linear growth and head growth in the higher‐protein group (Liu et al., 2015). Increased weight gain (MD 2.36 g/kg/day; 95% CI 1.3, 3.4) and head growth (MD 0.37 cm/week; 95% CI 0.16, 0.58) were also seen in a comparison of high‐ versus low‐protein formula (Fenton et al., 2014). One review of protein‐fortified infant milk, including both human milk and formula, found no difference in weight (MD 0.19 kg; 95% CI −0.03, 0.42) or length (MD 0.06 cm; 95% CI −0.22, 0.34) but a significant decrease in weight‐for‐age (MD −0.81; 95% CI −1.16, −0.46), length‐for‐age (MD −1.31; 95% CI −1.60, −1.01) and weight‐for‐length (MD −1.57; 95% CI −2.02, −1.12) *Z* scores of the protein‐fortified groups compared with unfortified or lower‐protein human milk (Pimpin et al., 2019).

Two reviews examining the effect of individual amino acid fortification found no difference in growth was observed (Cao et al., 2018; Moe‐Byrne et al., 2016). Taurine fortification in formula was associated with a decrease in linear growth (MD −0.18 cm/week; 95% CI −0.27, −0.09) but no effect on weight gain (MD 0.28 g/kg/day; 95% CI −0.47, 1.03) or head growth (MD 0.05 cm/week; 95% CI −0.06, 0.16) (Cao et al., 2018). For Moe‐Byrne et al., no meta‐analysis was conducted, but glutamine fortification was not found to affect weight gain in two of three primary studies described in the systematic review. One primary study was reported to show a positive association with weight gain, linear growth and head growth, although the review did not calculate an effect size (Moe‐Byrne et al., 2016).

### 3.4 Strength of evidence for individual meta‐analyses

Nineteen systematic reviews presented the data for a combined total of 100 meta‐analyses. We graded the quality of evidence of the individual meta‐analyses on the basis of established criteria commonly used in umbrella reviews as convincing, highly suggestive, suggestive, weak or nonsignificant (Belbasis et al., 2015; Bellou et al., 2017; Fusar‐Poli & Radua, 2018). See Data S1 for a complete list of meta‐analyses and associated strength of evidence components. No individual meta‐analysis met criteria of convincing or highly suggestive evidence. Seven associations were supported by suggestive evidence. These included greater weight gain, linear growth and head growth associated with (1) energy and protein‐fortified human milk compared with unfortified human milk and (2) formula (term or preterm) compared with donor human milk (fortified or unfortified), as well as greater weight gain associated with preterm formula compared with fortified donor human milk. Fifty meta‐analyses met criteria for weak evidence. Thirty‐nine meta‐analyses were not significant. Four meta‐analyses were not adequately assessed because of the absence of data.

## 4 DISCUSSION

### 4.1 Summary of main results

This umbrella review found 26 reviews composed of 150 unique primary studies evaluating the effect of infant milk options on the growth of LBW infants up to 6 months post‐term. We found evidence that energy and protein fortification of human milk is associated with increased weight gain, linear growth and head circumference compared with unfortified human milk (quality: suggestive) (Brown et al., 2016). We also found evidence that formula is associated with increased weight gain, linear growth and head growth compared with donor human milk (quality: suggestive) (Quigley et al., 2019).

These specific findings stand in contrast to the WHO's *Guidelines on Optimal Feeding for Low‐Birthweight Infants in Low‐ and Middle‐Income Countries* (2011), which recommend donor human milk over formula and fortification only in the case of growth failure. The WHO recommendations are strongly influenced by the protective effects of human milk against necrotizing enterocolitis. We acknowledge that many factors beyond a simple calculation of growth are important to consider in the formation of feeding recommendations, but our findings beg the question of what is the most appropriate feeding strategy for this population, particularly for infants with birthweight 1500 to <2500 g who are at lower risk for necrotizing enterocolitis than infants with birthweight <1500 g.

Multiple systematic reviews supported the use of higher protein content in both human milk and formula to increase growth, although the sample size was too small for this evidence to be considered ‘suggestive’, as defined by umbrella review criteria. Several interventions did not result in increased growth. These include infant milk with added fat, carbohydrate, LC‐PUFA, glutamine or taurine. Hydrolyzation also seemed to make no difference in growth. These interventions generally occurred during the birth hospitalization.

Several reviews investigated the question of postdischarge fortification of human milk and formula. This was generally not associated with increased anthropometric parameters at 3 to 4 months or 6 months post‐term (Young et al., 2013, 2016). This finding raises questions about the continued use of fortification after discharge, a common practice following the initial hospitalization of LBW infants. The use of energy and protein‐fortified formula may be less practical for many families because of limited access and increased cost of fortified formula compared with standard formula. Energy and protein‐fortified human milk requires the steps of expressing the milk, mixing it with milk fortifier, and bottle feeding an infant, a process that can be burdensome compared with direct breastfeeding.

It is important to acknowledge that we considered greater growth to be desirable in this population given the high risk for poor growth early in life and associated morbidities. There is increasing evidence that children who were born with LBW are at increased risk for metabolic syndrome later in life, particularly those who were SGA. The paradigm of ‘growth is good’ may not be appropriate in an older cohort. We attempted to include more nuanced measures of growth such as skinfold thickness or fat‐free mass but found little data in the review literature.

### 4.2 Quality of the evidence

We evaluated the quality of evidence for individual meta‐analyses included in this review based on extracted data. No meta‐analysis met the criteria for convincing evidence. The comparison of formula (term or preterm) with donor human milk (unfortified or fortified) by Quigley et al. (2019) would have been considered convincing if the fixed‐effects *P*‐value were <0.00001 instead of the actual value of exactly 0.00001, which downgraded the quality to ‘weak’. Seven meta‐analyses were considered suggestive. All other meta‐analyses for which sufficient information was available were considered to have weak quality of evidence or be nonsignificant. The quality of many meta‐analyses was downgraded because of the size of the review population. Many studies were small, possibly a reflection of the limitations inherent in conducting studies on LBW infants.

### 4.3 Completeness and applicability of the evidence

In addition to limitations due to the quality of evidence, the generalizability of the included reviews may be limited because of the population represented within the primary studies. Most reviews were primarily composed of studies of very low birthweight (VLBW) (birthweight <1500 g) preterm infants in high‐income countries.

Although we sought to include recommendations for all LBW infants, the study populations in the included reviews were concentrated within the VLBW weight band. Eighty‐one per cent of reviews had an average population birthweight between 1000 and 1500 g, whereas only 15% had an average birthweight falling between 1500 and <2500 g (Cao et al., 2018; Fenton et al., 2014; Gibson et al., 2001; Santiago et al., 2019). Most of these were deemed to be low quality on the basis of the AMSTAR 2 rating (Cao et al., 2018; Gibson et al., 2001; Santiago et al., 2019). This is significant because the growth patterns of VLBW infants cannot necessarily be extrapolated to infants within the higher birthweight population. Based on our findings, the unique population of infants with birthweight between 1500 and <2500 g is underrepresented in the current review literature. These infants constitute the majority of LBW infants, but the current WHO recommendations are based on literature with a population that is not truly representative of this contingent of the LBW population.

SGA full‐term infants were another LBW group that was underrepresented among these reviews. SGA infants have unique growth patterns and nutritional requirements (Tudehope et al., 2013). Only one low‐quality review specifically focused on full‐term infants who were SGA (Santiago et al., 2019). Nutritional recommendations for a VLBW preterm population cannot necessarily be extrapolated to the SGA full‐term infant. This is particularly important for an LMIC setting, in which the majority of LBW infants will be SGA but term (Lee et al., 2013).

We found that the majority of research on LBW feeding was conducted in high‐ or upper middle–income countries, which does not align with the greatest global prevalence of this population. Among the 121 studies with a location that was identified in the reviews, 118 were conducted in high‐ or upper middle–income countries or regions, and three studies were conducted in India, a lower middle–income country. No studies were identified with a setting in a low‐income country or from sub‐Saharan Africa. The majority of LBW infants are born in low‐ or lower middle–income countries, but these children are not well represented in the current body of research (Blencowe et al., 2019). The data regarding growth of infants in high‐income settings cannot necessarily be extrapolated to a low‐income population. For instance, studies of formula fortification in term children have demonstrated larger effects on weight and height in populations with a lower baseline nutritional status, suggesting that an at‐risk group may experience different growth patterns (Pimpin et al., 2019). More research is warranted on the optimal feeding of LBW infants within the context of low‐ and lower middle–income countries.

### 4.4 Limitations

The umbrella review methodology facilitates the evaluation of a broad research question in a manner difficult to achieve in an individual systematic review. We chose to conduct this type of review given the breadth of literature regarding nutrition of LBW infants. The scope that can be achieved is sweeping in nature; however, the overview methodology has a number of inherent limitations. Primary studies related to the research question but not included in other systematic reviews may be missed, potentially excluding valuable information. Umbrella reviews are necessarily limited to the most recent literature preceding the search date of the individual systematic reviews. The majority of reviews in our study were published in the past 5 years, but four reviews were >10 years old. We used a strength of evidence classification system that has been well described in the umbrella review literature, but which relies heavily upon *P*‐values. The *P*‐value is a potentially misleading tool for determining the quality of a study, as it does not account for risk of bias or the degree to which a significant result may be clinically meaningful. We have included the AMSTAR 2 results and the forest plans with effect size and 95% confidence intervals to provide a more complete picture of the quality and strength of the evidence for the individual systematic reviews and meta‐analyses included in this umbrella review.

Given the broad scope of interventions that we considered in this review, we limited our outcome of interest to infant growth. Necrotizing enterocolitis, neurodevelopment, kidney function and a number of other outcomes in preterm infants have all been correlated with the choice of infant milk and milk components. Choosing to focus on a single outcome, albeit an important one, provides an incomplete picture of the effect of nutrition on the heterogeneous components of health and well‐being.

We were interested in all metrics of growth but found the majority of growth outcomes were reported as weight gain, linear growth and head growth. Important measures of body composition, such as fat‐free body mass or skinfold thickness, received very little attention in the review literature.

## 5 CONCLUSION

Through this umbrella review, we were able to conduct a high‐level survey of the landscape of evidence on various sources of nutrition for LBW infants and their association with growth that led us to a number of conclusions.

1. Energy and protein fortification of human milk is associated with increased weight gain, linear growth and head growth (quality of evidence: suggestive).
2. Formula compared with donor human milk is associated with increased weight gain, linear growth and head growth (quality of evidence: suggestive).
3. Studies of the ideal nutritional interventions for LBW infants in low‐ and lower middle–income countries are vastly underrepresented in the literature.
4. Reviews of infant milk interventions are focused primarily on the <1500 g birthweight population, with few studies focused primarily on infants in the 1500 to <2500 g weight band, a group that may have a unique nutritional and growth profile.

Based on the gaps we have identified, we recommend additional research focused on the nutritional needs of infants with a birthweight 1500 to <2500 g and infants born in LMICs because both of these subgroups represent vulnerable populations who are underrepresented in the available review literature. We included all growth metrics in our search but found very few outcomes in the reviews addressing body composition or assessment of lean versus fat mass, important areas of focus for future research.

## CONFLICTS OF INTEREST

The authors declare that they have no conflicts of interest.

## CONTRIBUTIONS

KEAS, KN, MMD, CB, ACL, LV and LA contributed to the design of the study. KN, KEAS and MMD designed the literature search. KN and MMD screened records, extracted data and assessed risk of bias. KEAS adjudicated study selection. KN wrote the report. All authors provided critical conceptual input and critically revised the report.

## Supporting information


**Data S1.** Supporting information

## Data Availability

The data that support the findings of this study are available from the corresponding author upon reasonable request.
