# Supplementary material for: The effect of milk type and fortification on the growth of low‐birthweight infants: An umbrella review of systematic reviews and meta‐analyses
Source: Matern Child Nutr. 2021 Mar 17;17(3):e13176. doi: 10.1111/mcn.13176 (PMC8189224; doi:10.1111/mcn.13176)
Supplement: Supplementary file 1 — Data S1. Supporting information [file MCN-17-e13176-s001.docx]

**SUPPLEMENTARY MATERIAL**

**Appendix Table of Contents**

1. Sample keyword/search strategy
2. Data extraction list
3. List of excluded full-text studies with reason for exclusion
4. PRISMA checklist
5. Low-birthweight infant nutrition umbrella review protocol
6. Table: Detailed characteristics of included studies
7. Table: Primary studies included in multiple reviews
8. Table: Growth outcomes in individual reviews
9. Table: Strength of evidence of individual meta-analyses
10. **Sample keyword/search strategy:** PubMed search strategy

("Infant, Low Birth Weight"[Mesh] OR "Infant, Premature"[Mesh] OR low birth weight*[tiab] OR low birth weight[tiab] OR preterm[tiab] OR premature birth[tiab] OR premature infant*[tiab] OR premature newborn*[tiab] OR neonatal underweight[tiab] OR lbw infant*[tiab] OR lbw neonate*[tiab] OR small for gestational age[tiab] OR failure to thrive[tiab])

AND

("Infant Nutritional Physiological Phenomena"[mesh] OR "Infant Food"[Mesh] OR "Milk, Human"[mesh] OR breast feeding[tiab] OR breastfeeding[tiab] OR breastfed[tiab] OR breast fed[tiab] OR breast milk[tiab] OR breast milk[tiab] OR mother's own milk[tiab] OR mother's milk[tiab] OR infant nutrition*[tiab] OR neonatal nutrition*[tiab] OR infant feeding[tiab] OR neonatal feeding[tiab] OR infant formula*[tiab] OR bottle feed*[tiab] OR bottle fed[tiab] OR human donor milk[tiab] OR human milk[tiab])

AND

("Meta-Analysis as Topic"[Mesh] OR "Meta-Analysis"[Publication Type] OR systematic*[tiab] OR meta-analys*[tiab] OR metaanalys*[tiab] OR study selection[tiab] OR literature search*[tiab] OR database search*[tiab] OR electronic database*[tiab] OR pubmed[tiab] OR medline[tiab] OR embase[tiab] OR "Cochrane Database Syst Rev"[Journal] OR "Syst Rev"[Journal] OR "JBI Database System Rev Implement Rep"[Journal])

1. **Data extraction list**
2. Title
3. Author
4. Journal information
5. Year review published
6. Objectives
7. Databases searched
8. Participants (criteria for inclusion)
9. Feeding intervention
10. Control
11. Types of studies included
12. Total studies included
13. Total studies addressing the impact of infant milk/fortification on low-birthweight growth
14. Participants (# included)
15. Participants (with average birthweight in g)
16. Publication years of included studies
17. Country of origin
18. Appraisal instruments
19. Growth outcomes: weight, length, head circumference, other
20. Meta-analysis vs systematic review
21. Significance/direction
22. Heterogeneity
23. For each individual meta-analysis: summary random-effects P-value from test for overall effect, I^2^, population size, 95% confidence interval exclusion of the null
24. **List of excluded full-text studies with reason for exclusion**

**Wrong patient population**

Makrides, M., Gibson, R. A., Udell, T., Ried, K., & International LCPUFA Investigators. (2005). Supplementation of infant formula with long-chain polyunsaturated fatty acids does not influence the growth of term infants. *The American Journal of Clinical Nutrition*, *81*(5), 1094–1101. https://doi.org/10.1093/ajcn/81.5.1094

**Text not in English**

Aguilar Cordero, M. J., Sánchez López, A. M., Mur Villar, N., Hermoso Rodríguez, E., & Latorre García, J. (2014). [Effect of nutrition on growth and neurodevelopment in the preterm infant: a systematic review]. *Nutricion Hospitalaria : Organo Oficial de La Sociedad Espanola de Nutricion Parenteral y Enteral*, *31*(2), 716–729. <https://doi.org/10.3305/nh.2015.31.2.8266>

Fang, A., Chen, S., Han, J., & Zhu, H. (2018). Health effects of infant and follow-on formulas supplemented with long-chain polyunsaturated fatty acids: systematic review. *Acta Nutrimenta Sinica*, *40*(6), 531–543.

Fang, A., Wu, S., Han, J., & Zhu, H. (2018). Adequate protein content in infant and follow-on formulas: a systematic review. *Ying Yang Xue Bao] Acta Nutrimenta Sinica*, *41*(1), 7–16.

López-Torres, E., Doblas, P. A., Guerrero Del Valle, V., & De Linares, M. C. (2007). Clinical evaluation of omega-3 fatty acids on pregnancy, breast feeding, and infant development. *Clinica e Investigacion En Ginecologia y Obstetricia*, *34*(3), 100–105.

Martinez, F. E., & Camelo Jr, J. S. (2001). Nutrition of the preterm infants. *Jornal de Pediatria*, *77*(Suppl 1), S32-40.

Odièvre, M. H., & Olivier, C. (2002). Food and growth in premature infants. *Archives Ed Pediatrie*, *9*(Suppl 4), 459s–460s.

**Duplicate review published in a different journal**

Amissah, E., Brown, J., Crowther, C. A., & Harding, J. E. (2018). Protein supplementation of human milk for promoting growth in preterm infants: a Cochrane systematic review. *Journal of Paediatrics and Child Health*, *54*, 6–7. <https://doi.org/10.1111/jpc.13882_10>

Moon, K., Rao, S., Patole, S., Simmer, K., & Schulzke, S. (2017). Longchain polyunsaturated fatty acid supplementation in preterm infants: updated Cochrane review. *Journal of Paediatrics and Child Health*, *53*, 69–70.

**Non-protocol intervention**

Basuki, F., Hadiati, D. R., Turner, T., McDonald, S., & Hakimi, M. (2019). Dilute versus full-strength formula in exclusively formula-fed preterm or low birth weight infants. *Cochrane Database of Systematic Reviews*, *6*, CD007263. <https://doi.org/10.1002/14651858.CD007263.pub3>

Castanys-Muñoz, E., Kennedy, K., Castañeda-Gutiérrez, E., Forsyth, S., Godfrey, K. M., Koletzko, B., … Ong, K. K. (2017). Systematic review indicates postnatal growth in term infants born small-for-gestational-age being associated with later neurocognitive and metabolic outcomes. *Acta Paediatrica*, *106*(8), 1230–1238. <https://doi.org/10.1111/apa.13868>

Mimouni, F. B., Nathan, N., Ziegler, E. E., Lubetzky, R., & Mandel, D. (2017). The use of multinutrient human milk fortifiers in preterm infants: a systematic review of unanswered questions. *Clinics in Perinatology*, *44*(1), 173–178. <https://doi.org/10.1016/j.clp.2016.11.011>

Ross, E. S., & Browne, J. V. (2013). Feeding outcomes in preterm infants after discharge from the neonatal intensive care unit (NICU): a systematic review. *Newborn and Infant Nursing Reviews*, *13*(2), 87–93. <https://doi.org/10.1053/j.nainr.2013.04.003>

**Wrong study design**

Ben, X.-M. (2008). Nutritional management of newborn infants: practical guidelines. *World Journal of Gastroenterology*, *14*(40), 6133–6139.

Hale, J. R. (2014). Dilute versus full-strength formula in exclusively formula-fed preterm or low-birth-weight infants: a summary of findings from the Cochrane Library with implications for critical care nursing. *Critical Care Nurse*, *34*(6), 70–72. <https://doi.org/10.4037/ccn2014741>

McLeod, G., & Sherriff, J. (2007). Preventing postnatal growth failure--the significance of feeding when the preterm infant is clinically stable. *Early Human Development*, *83*(10), 659–665. <https://doi.org/10.1016/j.earlhumdev.2007.07.010>

Wagner, J., Hanson, C., & Anderson-Berry, A. (2014). Considerations in meeting protein needs of the human milk-fed preterm infant. *Advances in Neonatal Care*, *14*(4), 281–289. <https://doi.org/10.1097/ANC.0000000000000108>

**No eligible studies**

Brown, J. V. E., Walsh, V., & McGuire, W. (2019). Formula versus maternal breast milk for feeding preterm or low birth weight infants. *Cochrane Database of Systematic Reviews*, *8*, CD002972. https://doi.org/10.1002/14651858.CD002972.pub3

Dempsey, E., & Miletin, J. (2019). Banked preterm versus banked term human milk to promote growth and development in very low birth weight infants. *Cochrane Database of Systematic Reviews*, *6*, CD007644. <https://doi.org/10.1002/14651858.CD007644.pub3>

Henderson, G., Anthony, M. Y., & McGuire, W. (2007). Formula milk versus maternal breast milk for feeding preterm or low birth weight infants. *Cochrane Database of Systematic Reviews*, (4), CD002972. <https://doi.org/10.1002/14651858.CD002972.pub2>

Henderson, G., Fahey, T., & McGuire, W. (2007b). Nutrient-enriched formula milk versus human breast milk for preterm infants following hospital discharge. *Cochrane Database of Systematic Reviews*, (4), CD004862. https://doi.org/10.1002/14651858.CD004862.pub2

**Full text not available**

Garcia-Stewart, S., & Mitchell, S. A. (2013). Mothers’ own milk for the feeding of preterm infants: a systematic literature review. *Value in Health*, *16*(7), A330. <https://doi.org/10.1016/j.jval.2013.08.048>

Miller, J., Makrides, M., & Collins, C. T. (2008). High versus standard protein content of human milk fortifier for promoting growth and neurological development in preterm infants. *Cochrane Database of Systematic Reviews*. <https://doi.org/10.1002/14651858.CD007090>

**Updated review available**

Basuki, F., Hadiati, D. R., Turner, T., McDonald, S., & Hakimi, M. (2013). Dilute versus full strength formula in exclusively formula-fed preterm or low birth weight infants. *Cochrane Database of Systematic Reviews*, (11), CD007263. https://doi.org/10.1002/14651858.CD007263.pub2

Henderson, G., Fahey, T., & McGuire, W. (2007a). Multicomponent fortification of human breast milk for preterm infants following hospital discharge. *Cochrane Database of Systematic Reviews*, (4), CD004866. <https://doi.org/10.1002/14651858.CD004866.pub2>

McGuire, W., & Anthony, M. Y. (2001). Formula milk versus preterm human milk for feeding preterm or low birth weight infants. *Cochrane Database of Systematic Reviews*, (3), CD002972. <https://doi.org/10.1002/14651858.CD002972>

Ng, D. H. C., Klassen, J., Embleton, N. D., & McGuire, W. (2017). Protein hydrolysate versus standard formula for preterm infants. *Cochrane Database of Systematic Reviews*, *10*, CD012412. https://doi.org/10.1002/14651858.CD012412.pub2

Quigley, M., Embleton, N. D., & McGuire, W. (2018). Formula versus donor breast milk for feeding preterm or low birth weight infants. *Cochrane Database of Systematic Reviews*, *6*, CD002971. <https://doi.org/10.1002/14651858.CD002971.pub4>

Verner, A., Craig, S., & McGuire, W. (2007). Effect of taurine supplementation on growth and development in preterm or low birth weight infants. *Cochrane Database of Systematic Reviews*, (4), CD006072. <https://doi.org/10.1002/14651858.CD006072.pub2>

**Wrong outcomes**

Lin, L., Amissah, E., Gamble, G. D., Crowther, C. A., & Harding, J. E. (2019). Impact of macronutrient supplements for children born preterm or small for gestational age on developmental and metabolic outcomes: a systematic review and meta-analysis. *PLoS Medicine*, *16*(10), e1002952. https://doi.org/10.1371/journal.pmed.1002952

Rosenfeld, E., Beyerlein, A., Hadders-Algra, M., Kennedy, K., Singhal, A., Fewtrell, M., … von Kries, R. (2009). IPD meta-analysis shows no effect of LC-PUFA supplementation on infant growth at 18 months. *Acta Paediatrica*, *98*(1), 91–97. https://doi.org/10.1111/j.1651-2227.2008.00988.x

Szajewska, H. (2007). Extensive and partial protein hydrolysate preterm formulas. *Journal of Pediatric Gastroenterology and Nutrition*, *45 Suppl 3*, S183-7. <https://doi.org/10.1097/01.mpg.0000302969.60373.39>

Zhang, P., Lavoie, P. M., Lacaze-Masmonteil, T., Rhainds, M., & Marc, I. (2014). Omega-3 long-chain polyunsaturated fatty acids for extremely preterm infants: a systematic review. *Pediatrics*, *134*(1), 120–134. https://doi.org/10.1542/peds.2014-0459

Long Chain polyunsaturated fatty acid supplementation in preterm infants: updated Cochrane Review. (2017). *Journal of Paediatrics and Child Health*, *53*, 69–70. https://doi.org/10.1111/jpc.13494_204

1. **PRISMA checklist**

| **Section/topic** | **#** | **Checklist item** | **Reported on page #** |
| --- | --- | --- | --- |
| **TITLE** | | | |
| Title | 1 | Identify the report as a systematic review, meta-analysis, or both. | 1 |
| **ABSTRACT** | | | |
| Structured summary | 2 | Provide a structured summary including, as applicable: background; objectives; data sources; study eligibility criteria, participants, and interventions; study appraisal and synthesis methods; results; limitations; conclusions and implications of key findings; systematic review registration number. | 1 |
| **INTRODUCTION** | | | |
| Rationale | 3 | Describe the rationale for the review in the context of what is already known. | 2 |
| Objectives | 4 | Provide an explicit statement of questions being addressed with reference to participants, interventions, comparisons, outcomes, and study design (PICOS). | 3 |
| **METHODS** | | | |
| Protocol and registration | 5 | Indicate if a review protocol exists, if and where it can be accessed (e.g., Web address), and, if available, provide registration information including registration number. | 4 |
| Eligibility criteria | 6 | Specify study characteristics (e.g., PICOS, length of follow-up) and report characteristics (e.g., years considered, language, publication status) used as criteria for eligibility, giving rationale. | 4 |
| Information sources | 7 | Describe all information sources (e.g., databases with dates of coverage, contact with study authors to identify additional studies) in the search and date last searched. | 4 |
| Search | 8 | Present full electronic search strategy for at least one database, including any limits used, such that it could be repeated. | 4, Appendix |
| Study selection | 9 | State the process for selecting studies (i.e., screening, eligibility, included in systematic review, and, if applicable, included in the meta-analysis). | 5 |
| Data collection process | 10 | Describe method of data extraction from reports (e.g., piloted forms, independently, in duplicate) and any processes for obtaining and confirming data from investigators. | 5 |
| Data items | 11 | List and define all variables for which data were sought (e.g., PICOS, funding sources) and any assumptions and simplifications made. | 5, Appendix |
| Risk of bias in individual studies | 12 | Describe methods used for assessing risk of bias of individual studies (including specification of whether this was done at the study or outcome level), and how this information is to be used in any data synthesis. | 6 |
| Summary measures | 13 | State the principal summary measures (e.g., risk ratio, difference in means). | N/A |
| Synthesis of results | 14 | Describe the methods of handling data and combining results of studies, if done, including measures of consistency (e.g., I^2^) for each meta-analysis. | N/A |
| Risk of bias across studies | 15 | Specify any assessment of risk of bias that may affect the cumulative evidence (e.g., publication bias, selective reporting within studies). | 6 |
| Additional analyses | 16 | Describe methods of additional analyses (e.g., sensitivity or subgroup analyses, meta-regression), if done, indicating which were prespecified. | 6 |
| **RESULTS** | | | |
| Study selection | 17 | Give numbers of studies screened, assessed for eligibility, and included in the review, with reasons for exclusions at each stage, ideally with a flow diagram. | 6-7 |
| Study characteristics | 18 | For each study, present characteristics for which data were extracted (e.g., study size, PICOS, follow-up period) and provide the citations. | 8-10 |
| Risk of bias within studies | 19 | Present data on risk of bias of each study and, if available, any outcome-level assessment (see Item 12). | 11, 16 |
| Results of individual studies | 20 | For all outcomes considered (benefits or harms), present, for each study: (a) simple summary data for each intervention group and (b) effect estimates and confidence intervals, ideally with a forest plot. | 17, Appendix |
| Synthesis of results | 21 | Present results of each meta-analysis done, including confidence intervals and measures of consistency. | N/A |
| Risk of bias across studies | 22 | Present results of any assessment of risk of bias across studies (see Item 15). | 16 |
| Additional analysis | 23 | Give results of additional analyses, if done (e.g., sensitivity or subgroup analyses, meta-regression [see Item 16]). | N/A |
| **DISCUSSION** | | | |
| Summary of evidence | 24 | Summarize the main findings including the strength of evidence for each main outcome; consider their relevance to key groups (e.g., health care providers, users, and policy makers). | 18 |
| Limitations | 25 | Discuss limitations at study and outcome level (e.g., risk of bias) and at review level (e.g., incomplete retrieval of identified research, reporting bias). | 22 |
| Conclusions | 26 | Provide a general interpretation of the results in the context of other evidence, and implications for future research. | 23 |
| **FUNDING** | | | |
| Funding | 27 | Describe sources of funding for the systematic review and other support (e.g., supply of data); role of funders for the systematic review. | 24 |

1. **Low-birthweight infant nutrition umbrella review protocol**
2. Background and aims
   1. Background

PICO (see II.B. below for more detail)

- - - 1. P (population): meta-analyses/systematic reviews of low-birthweight infants
      2. I (intervention): feeding options used as alternatives to unfortified maternal breast milk, including both milk fortification with macronutrients and breast milk substitutes (donor human milk, formula, etc)
      3. C (comparison/control): other feeding options or unfortified maternal breast milk
      4. O (outcome): growth
  1. Review question: How does the growth of low-birthweight infants who are fed with alternatives to unfortified maternal breast milk, including both breastmilk substitutes and milk fortification, compare with that of those who are fed either unfortified maternal breast milk or any of the other feeding options?

1. Methods
   1. Search Strategy
      1. We will search the following electronic bibliographic databases: PubMed and EMBASE, CINAHL, and Web of Science.
      2. The search strategy will include only terms relating to or describing the population, intervention, and type of study. Since this is an umbrella review, we are limiting our search to previously published meta-analyses and systematic reviews. We will not include umbrella reviews. In the case of reviews for which updated versions are available, only the most recent version will be included. We will restrict our search to reviews that are published in English. Please see attachment for full search strategy.
      3. Language: We will restrict our search to reviews that are published in English.
      4. URL: An example URL for the search strategy used in Pubmed is [here](https://www.ncbi.nlm.nih.gov/pubmed/?term=(%22Infant%2C+Low+Birth+Weight%22%5BMesh%5D+OR+%22Infant%2C+Premature%22%5BMesh%5D+OR+low+birth+weight*%5Btiab%5D+OR+low+birthweight%5Btiab%5D+OR+preterm%5Btiab%5D+OR+premature+birth%5Btiab%5D+OR+premature+infant*%5Btiab%5D+OR+premature+newborn*%5Btiab%5D+OR+neonatal+underweight%5Btiab%5D+OR+lbw+infant*%5Btiab%5D+OR+lbw+neonate*%5Btiab%5D+OR+small+for+gestational+age%5Btiab%5D+OR+failure+to+thrive%5Btiab%5D)+AND+(%22Infant+Nutritional+Physiological+Phenomena%22%5Bmesh%5D+OR+%22Infant+Food%22%5BMesh%5D+OR+%22Milk%2C+Human%22%5Bmesh%5D+OR+breast+feeding%5Btiab%5D+OR+breastfeeding%5Btiab%5D+OR+breastfed%5Btiab%5D+OR+breast+fed%5Btiab%5D+OR+breast+milk%5Btiab%5D+OR+breastmilk%5Btiab%5D+OR+mother%27s+own+milk%5Btiab%5D+OR+mother%27s+milk%5Btiab%5D+OR+infant+nutrition*%5Btiab%5D+OR+neonatal+nutrition*%5Btiab%5D+OR+infant+feeding%5Btiab%5D+OR+neonatal+feeding%5Btiab%5D+OR+infant+formula*%5Btiab%5D+OR+bottle+feed*%5Btiab%5D+OR+bottle+fed%5Btiab%5D+OR+human+donor+milk%5Btiab%5D+OR+human+milk%5Btiab%5D)+AND+(%22Meta-Analysis+as+Topic%22%5BMesh%5D+OR+%22Meta-Analysis%22%5BPublication+Type%5D+OR+systematic*%5Btiab%5D+OR+meta-analys*%5Btiab%5D+OR+metaanalys*%5Btiab%5D+OR+study+selection%5Btiab%5D+OR+literature+search*%5Btiab%5D+OR+database+search*%5Btiab%5D+OR+electronic+database*%5Btiab%5D+OR+PubMed%5Btiab%5D+OR+MEDLINE%5Btiab%5D+OR+embase%5Btiab%5D+OR+%22Cochrane+Database+Syst+Rev%22%5BJournal%5D+OR+%22Syst+Rev%22%5BJournal%5D+OR+%22JBI+Database+System+Rev+Implement+Rep%22%5BJournal%5D)).
   2. Objectives/detailed PICO
      1. Domain: Low-birthweight infant feeding
      2. Population:
         1. Inclusion: Infants born with low birthweight (<2500 g).This may include infants who are of term gestation and small for gestational age or infants who are premature.
         2. Exclusion: Systematic reviews of infants with a birthweight >2500 g. If the birthweight is not explicitly stated in the inclusion criteria, we will calculate the weighted average birthweight of infants from the primary studies and use this to determine whether a systematic review is eligible for inclusion. Because this is a review of reviews, we anticipate that some reviews may include subjects who overlap with our goal population while also including subjects outside of our predefined parameters. If a review identifies separate subpopulations for meta-analysis, we will consider these subpopulations independently.
            1. Birthweight:

Some of the work that has been published in this area is based on gestational age classification as opposed to birthweight. For studies in which gestational age but not birthweight is reported, we will calculate the birthweight that is at the 50th percentile on the Intergrowth curve for the average gestational age and use that as a proxy for average birthweight unless the study otherwise indicates that a large proportion of the population was either small or large for gestational age.

For studies in which a range of eligible birthweights is included but no average birthweight or gestational age is presented, we will use the highest included value as a proxy for average birthweight so as to be overly inclusive. As we are only considering growth as an outcome, primary studies with no growth metrics reported will not be included in the calculations of average birthweight.

- - - 1. Intervention: We will examine infant feeding options that are used as alternatives to unfortified maternal breast milk, including donor human milk, term formula, preterm formula, and milk fortifiers. We will also consider systematic reviews addressing macronutrient (fat, protein, and carbohydrate) components of any of these milk options including their specific types and compositions, i.e., specific fatty acids, amino acids, or lactose content components. We will not examine micronutrient supplementation. We will also exclude reviews of methods of feeding such as feeding from the breast vs bottle.
    1. Comparison: The comparison will be conducted among infant feeding options that include unfortified and fortified maternal breast milk, unfortified and fortified donor human milk, and infant formula. Comparison may also be conducted among macronutrient supplementation methods such as protein or fat supplements.
    2. Types of study to be included: We plan to include meta-analyses and systematic reviews (using PRISMA or other internationally accepted methodologies) within the scope of our project. We will exclude reviews that incorporate theoretical studies or opinion as their primary source of evidence. We will exclude other umbrella reviews or studies found only in grey literature.
    3. Context: We do not plan to limit studies to a specific context. We plan to include both hospital- and community-based feeding strategies in our study.
    4. Main outcome: Our primary outcome will be infant growth up to 6 months of age. We will consider growth metrics that include weight, length, and head circumference velocities and Z scores, body composition, and bone mass accretion. We will not consider other health outcomes, such as mortality, necrotizing enterocolitis, or neurodevelopment. We will plan to report both adverse and beneficial outcomes.
    5. Additional outcomes: None
  1. Study selection

Titles and/or abstracts of studies retrieved with the use of the search strategy will be screened independently by two review authors to identify studies that meet the inclusion criteria. The full text will be obtained for the potentially eligible studies and will be screened independently by the two review authors. We will use a review software system, Covidence, to upload and screen articles. A third review author will act as an adjudicator for discrepancies between the two primary reviewers. The review authors will not be blinded to author or journal during the study selection process.

- 1. Data extraction

A standardized, pre-piloted data extraction form will be used to pull data from the selected studies. We plan to use the JBI Umbrella Review Data Extraction Form as a template for building our data extraction form. Data entry will be completed with the use of the Covidence software system. Extracted information will include: title, author/year, objectives, type of review, participant (including gestational age and/or birthweight), setting/context, description of feeding interventions, number of databases searched, range of year in included studies, number of studies included, types of studies included, country of origin of included studies, appraisal instruments used, appraisal rating, method of analysis, outcome assessment, results/findings, significance/direction, heterogeneity, and additional comments. The data extraction forms will be completed independently by the two review authors and compared for discrepancies, which will be resolved through discussion and/or adjudication by a third review author. Authors of eligible studies will not be contacted to provide missing or additional data. Identification of inclusion/exclusion criteria will be based on the information provided in the systematic reviews and meta-analyses as opposed to those in the individual studies.

- - 1. Risk of bias assessment

The risk of bias will be evaluated in included studies by two independent review authors using the AMSTAR 2 checklist quality assessment tool. Disagreements between the two reviewers will be resolved by discussion and/or adjudication by a third reviewer.

- 1. Strategy for data synthesis
     1. Quantitative vs narrative synthesis: We plan to provide a narrative synthesis of the findings as well as tabular presentation of the data. Our data synthesis will be structured around the type of feeding intervention, the target population characteristics, the intervention context, and the outcome of infant growth. We will plan to present the number of studies informing the outcomes, the number of participants, and the heterogeneity of the results. We will also indicate which, if any, primary studies are included in more than one systematic review or meta-analysis. We anticipate that our tabular data will be organized into a table of included review characteristics and a table of findings.
     2. Analysis of subgroups or subsets
        1. We plan to subcategorize the data into studies from high-income countries vs low- or middle-income countries.
        2. If possible, we will conduct a subanalysis of infants <1500 and 1500–<2500 g.
  2. Other PROSPERO registration details
     1. Type and method of review: umbrella review
     2. Language: English
     3. Country: USA
     4. Other registration details: N/A
     5. Reference and/or URL for published protocol: N/A
     6. Dissemination plans: In addition to producing a report for the funders of this review, a paper will be submitted to a journal in this field.
     7. Keywords: umbrella review, systematic review, meta-analysis, low birthweight, premature, feeding, nutrition, growth, formula, breast milk, fortification, donor human milk
     8. Existing review of same topic by same authors: none
     9. Current review status: preliminary searches complete, data extraction not initiated
     10. Additional information: This review is being undertaken as part of the evidence-gathering process for a larger mixed-methods observational study to collect information on the state of low-birthweight feeding in low- or middle-income countries.

1. Table A1. Detailed characteristics of included studies

| **Author/Year** | **Type of milk** | **Types of included studies** | **# of relevant studies/participants** | **Weighted average birthweight** | **Publication years** |
| --- | --- | --- | --- | --- | --- |
| **Donor human milk** | | |  |  |  |
| Boyd 2007 | Donor human milk | RCTs, quasi-RCTs, and observational studies | 6/775 | 1481 | 1976–1994 |
| Quigley 2019 | Donor human milk | Randomized or quasi-RCTs | 11/1503 | 1257 | 1976–2018 |
| Yu 2019 | Donor human milk | RCTs | 4/307 | 1042 | 1983–2013 |
| Exclusive breastfeeding | | | | | |
| Santiago 2019 | Exclusive human breastfeeding | RCTs and case-control studies | 7 studies (5 cohorts)/502 | 2482 | 2006–2015 |
| Energy and protein supplementation | | | | | |
| Brown 2016 | Energy and protein fortification vs human milk | Randomized and quasi‐RCTs, including cluster RCTs | 14/1071 | 1268 | 1986–2012 |
| Walsh 2019 | Energy and protein fortification vs formula | Controlled trials using random or quasi‐random patient allocation | 7/590 | 1420 | 1984–1992 |
| Young 2013 | Energy and protein fortification vs postdischarge human milk | Controlled trials using random or quasi‐random patient allocation | 2/246 | 1258 | 2008–2011 |
| Young 2016 | Energy and protein fortification vs postdischarge formula | Controlled trials using random or quasi‐random patient allocation, including cluster RCTs | 16/1436 | 1243 | 1992–2012 |
| Teller 2016 | Energy and protein fortification vs postdischarge formula | Both RCTs and observational studies | 31/1089 | 1244 | 1985–2011 |
| Carbohydrate supplementation | | | | | |
| Amissah 2018 | Carbohydrate supplementation vs human milk | Randomized or quasi-RCTs | 1/75 | 1224 | 2014 |
| Fat supplementation | | | | | |
| Amissah 2018 | Fat supplementation vs formula | Published and unpublished controlled trials utilizing either random or quasi-random patient allocation | 1/14 | 1243 | 1989 |
| Gibson 2001 | Long-chain polyunsaturated fatty acids vs formula | RCTs | 11/1016 | 1536 | 1990–2000 |
| Moon 2016 | Long-chain polyunsaturated fatty acids vs formula | RCTs | 15/2246 | 1326 | 1990–2007 |
| Nehra 2002 | Medium-chain triglycerides vs formula | RCTs | 8/206 | 1253 | 1982–1993 |
| Newberry 2016 | Omega-3 long-chain polyunsaturated fatty acids vs formula | RCTs, prospective cohort and nested case control studies | 6/1511 | 1262 | 2005–2011 |
| Rodriguez 2012 | Omega-3 long-chain polyunsaturated fatty acids vs formula | RCTs and observational studies | 3/317 | 1364 | 1999–2010 |
| Udell 2005 | Alpha-linolenic acid and linolenic acid omega-3 long-chain polyunsaturated fatty acids vs formula | RCTs | 3/225 | 1496 | 1990–1997 |
| Protein supplementation | | | | | |
| Amissah 2018 | Protein supplementation vs human milk | Published and unpublished randomized and quasi‐RCTs | 6/204 | 1274 | 1982–2001 |
| Cao 2018 | Taurine vs formula | RCTs | 9/216 | 1815 | 1983–1993 |
| Fenton 2014 | Protein supplementation vs formula | RCTs | 6 (a priori), (10 post facto)/239 (a priori), 717 (post facto) | 1616 | 1969–2006 |
| Liu 2015 | Protein supplementation vs human milk | RCTs and prospective observational intervention studies | 5/352 | 1090 | 2000–2013 |
| Moe-Byrne 2016 | Glutamine | Controlled trials using random or quasi‐random participant allocation. Due to the nature of the intervention, we excluded cross‐over trials. | 4/893 | 958 | 1997–2007 |
| Pimpin 2019 | Protein supplementation vs formula or human milk | RCTs | 6/418 | 1332 | 2001–2010 |
| Tonkin 2014 | Protein supplementation vs formula or human milk | Randomized or quasi-RCTs | 24/987 | 1327 | 1976–2012 |
| Hydrolyzation | | | | | |
| Ng 2019 | Protein hydrolyzed formula | Randomized or quasi‐RCTs, including cluster RCTs | 7/424 | 1188 | 1992–2009 |
| Tan-Dy 2013 | Lactase-treated formula or human milk | Randomized or quasi-RCTs | 1/130 | 1394 | 2002 |

Abbreviations: RCTs, randomized controlled trials.

(7) Table A2. Primary studies included in multiple reviews

| **Primary studies** | **Systematic reviews** |
| --- | --- |
| Agosti 2003 | Teller 2016, Young 2016 |
| Bhatia 1991 | Teller 2016, Tonkin 2014 |
| Carver 2001 | Teller 2016, Young 2016 |
| Cooke 2006 | Cao 2018, Pimpin 2019 |
| DeCurtis 2002 | Teller 2016, Young 2016 |
| Diersen-Schade 1999 | Gibson 2001, Moon 2016 |
| Embleton 2005 | Fenton 2014, Pimpin 2019 |
| Faerk 2000 | Amissah 2018, Brown 2016 |
| Groh-Wargo 2005 | Moon 2016, Newberry 2016, Rodriguez 2012 |
| Gross 1983 | Boyd 2007, Brown 2016 |
| Innis 2002 | Moon 2016, Rodriguez 2012 |
| Jeon 2011 | Teller 2016, Young 2016 |
| Kashyap 1986 | Walsh 2019, Fenton 2014, Tonkin 2014 |
| Kashyap 1988 | Fenton 2014, Tonkin 2014 |
| Koo 2006 | Teller 2016, Young 2016 |
| Lucas 1992 | Teller 2016, Young 2016 |
| Lucas 2001 | Teller 2016, Young 2016 |
| O'Conner 2016 | Moon 2016, Young 2013 |
| Okamoto 1984 | Nehra 2002, Cao 2018 |
| Peng 2004 | Teller 2016, Young 2016 |
| Polberger 1989 | Amissah 2018 (protein), Brown 2016, Amissah 2018 (energy and protein) |
| Porcelli 1992 | Liu 2015, Brown 2016, Tonkin 2014 |
| Putet 2012 | Amissah 2018, Boyd 2007, Yu 2019 |
| Raiha 1972 | Boyd 2007, Fenton 2014 |
| Raiha 1976 | Quigley 2019, Tonkin 2014 |
| Roggero 2011 | Teller 2016, Young 2016 |
| Roggero 2012 | Teller 2016, Young 2016 |
| Ryan 1999 | Gibson 2001, Rodriguez 2012 |
| Taroni 2009 | Teller 2016, Young 2016 |
| Tyson 1989 | Cao 2018, Yu 2019 |
| Uauy 1990 | Udell 2005, Moon 2016 |
| Vanderhoof 1999 | Gibson 2001, Moon 2016 |
| Wauben 2005 | Brown 2016, Fenton 2014 |

(8) Table A3. Growth outcomes of individual reviews

|  | **Author/Year** | **Review type** | **Weight** | **Length** | **Head circumference** | **Other growth outcomes** |
| --- | --- | --- | --- | --- | --- | --- |
| DHM | | | | | | |
|  | Boyd 2007 | SR | **Formula vs DHM, sole diet** Trend toward more weight gain in formula group. Nine of 13 comparisons of early postnatal weight gain were significantly in favor of formula, 1 was significantly in favor of donor milk, and 3 demonstrated no significant difference.  **Formula vs DHM, supplementing mother's own milk** Weight significantly greater in formula group vs donor milk (1 study). | **Formula vs DHM** Trend toward length gain greater in formula vs donor group (significant in 5 comparisons). | **Formula vs DHM, sole diet** Trend toward more head circumference growth in formula vs donor milk, but only statistically significant in 1 of 5 comparisons. **Formula vs DHM, supplementing mother's milk** Head circumference significantly greater in formula group vs donor milk in 1 study. | **Formula vs DHM, sole diet** Skinfold thickness: Significantly greater gains in triceps and subscapular skinfold thickness in the formula vs DHM groups (2 comparisons in 1 study).  **Formula vs DHM, supplementing mother's milk** Skinfold thickness significantly greater in formula group vs donor milk (1 study). |
|  | Quigley 2019 | MA | **Formula vs DHM** MD 2.51 g/kg/d (1.93 to 3.08); 9 studies/1028 participants Time to regain birth weight: -3.08 d (-4.38, -1.77); 3 studies/236 participants  Subgroup analyses: **Term formula vs unfortified DHM** MD 1.74 g/kg/d (0.96, 2.53); 3 studies/234 participants **Preterm formula vs unfortified DHM** MD 4.16 (3.04, 5.28); 3 studies/249 participants **Preterm formula vs fortified DHM** MD 2.37 (1.09,3.65); 3 studies/545 participants  **Formula vs DHM, sole diet** MD 2.65 g/kg/d (1.94, 3.36); 6 studies/421 participants **Formula vs DHM, supplemental diet** MD 2.22 g/kg/d (1.23, 3.21); 3 studies/607 participants | **Formula vs DHM** Crown-heel length: MD 1.21 mm/wk (0.77, 1.65); 8 studies/820 participants Crown-rump length: MD 0.59 mm/wk (0.08, 1.10); 1 study/106 participants Femoral length: MD 0.34 mm/wk (0.13, 0.55); 1 study/106 participants  Subgroup analyses: **Term formula vs unfortified DHM** Crown-heel length: MD 0.80 mm/wk (0.10, 1.50); 2 studies/128 participants **Preterm formula vs unfortified DHM** Crown-heel length: MD 1.96 mm/wk (1.10, 2.82); 3 studies/147 participants **Preterm formula vs fortified DHM** Crown-heel length: MD 1.10 mm/wk (0.33, 1.87); 3 studies/545 participants  **Formula vs DHM, sole diet** Crown-heel length: MD 1.54 mm/wk (0.98, 2.11); 5 studies/283 participants **Formula vs DHM, supplemental diet** Crown-heel length: MD 0.67 mm/wk (-0.04, 1.38); 3 studies/537 participants | **Formula vs DHM** MD 0.85 mm/wk (0.47, 1.23); 8 studies/894 participants  Subgroup analyses: **Term formula vs unfortified DHM** MD 0.81 mm/wk (0.15, 1.47); 2 studies/128 participants **Preterm formula vs unfortified DHM** MD 2.01 (1.21, 2.81); 3 studies/221 participants **Preterm formula vs fortified DHM** MD 0.30 (-0.27, 0.86); 3 studies/545 participants  **Formula vs DHM, sole diet** MD 1.36 mm/wk (0.85,1.88); 5 studies/305 participants **Formula vs DHM, supplemental diet** MD 0.24 mm/wk (-0.32, 0.80); 3 studies/589 participants | No outcomes reported |
|  | Yu 2019 | MA | **DHM vs formula** MD -6.58 g/d (-11.19, -1.98); 4 studies/523 participants  Subgroup analysis: **Infants with BW <1000 g:** MD -2.80 g/d (-4.39, -1.20); 2 studies/219 participants **Infants with BW 1000-1500 g:** MD -10.42 g/d (-12.30, -8.53); 2 studies/88 participants | **DHM vs formula** MD -0.30 cm/wk (-0.41, -0.20); 3 studies/307 participants | **DHM vs formula** MD -0.16 cm/wk (-0.33, 0.01); 4 studies/206 participants  Subgroup analysis: **Infants with BW <1000 g:** MD -0.08 cm/wk (-0.19, 0.03); 2 studies/219 participants **Infants with BW 1000-1500 g**: MD -0.25 cm/wk (-0.55, 0.04); 2 studies/88 participants | No outcomes reported |
| Exclusive breastfeeding | | | | | | |
|  | Santiago 2019 | SR | Exclusive human milk feeding vs alternative infant feeding options Mixed results between studies with regard to weight gain in children fed human milk, fortified human milk, preterm formula, and term formula. | **Exclusive human milk feeding vs alternative infant feeding options** Description of mixed results in linear growth between children fed human milk, fortified human milk, preterm formula, and term formula. Significantly increased growth in infants fed fortified vs unfortified human milk. | No outcomes reported | **Exclusive human milk feeding vs alternative infant feeding options** Body composition by DEXA: Significantly lower fat mass but no difference in lean mass in breastfed vs preterm formula group at 4 months. No difference in lean or fat mass at 2 weeks or 4 months in breastfed term formula-fed groups. |
| Energy and protein supplementation | | | | | | |
|  | Brown 2016 | MA | **Human milk fortified with both energy (carbohydrate or fat) and protein vs unfortified human milk** MD 1.81 g/kg/d (1.23, 2.40); 10 studies/635 participants  Subgroup analysis: Trials recruiting only very preterm or VLBW infants: MD 2.82 g/kg/d (1.23, 2.40); 5 studies/269 participants  Trials conducted in low- or middle-income countries: MD 1.86 g/kg/d (0.70, 3.01); 2 studies/214 participants | **Human milk fortified with both energy (carbohydrate or fat) and protein vs unfortified human milk**  MD 0.18 cm/wk (0.10, 0.26); 8 studies/555 participants  Subgroup analysis: Trials recruiting only very preterm or VLBW infants: MD 0.21 cm/wk (0.14, 0.28); 3 studies/189 participants | **Human milk fortified with both energy (carbohydrate or fat) and protein vs unfortified human milk** MD 0.08 cm/wk (0.04, 0.12); 8 studies/555 participants  Subgroup analysis: Trials recruiting only very preterm or VLBW infants: MD 0.11 cm/wk (0.05, 0.17); 3 studies/189 participants | No outcomes reported |
|  | Teller 2016 | SR | **Postdischarge formula vs preterm formula vs standard term formula** All studies of enriched standard term formula demonstrated similar growth to nonenriched standard term formula. Postdischarge formula had mixed results compared with standard term formula. Most studies showed that preterm formula was associated with positive growth vs standard term formula. Most studies showed that enriched postdischarge formula showed similar weight gain to control. | **Postdischarge formula vs preterm formula vs standard term formula** Most studies of enriched standard term formula and postdischarge formula demonstrated similar growth as standard term formula. All studies showed similar length gain in enriched postdischarge formula compared with controls. | **Postdischarge formula vs preterm formula vs standard term formula** Most studies of enriched standard term formula and postdischarge formula demonstrated similar growth standard term formula. Most studies showed preterm formula was associated with positive head growth vs standard term formula. | No outcomes reported |
|  | Walsh 2019 | MA | **Nutrient-enriched vs standard formula** MD 2.43 g/kg/d (1.60, 3.26); 6 studies/440 participants Days to regain birthweight: MD 1.48 days (-4.73, 1.77); 3 studies/74 participants  Subgroup analysis:  **Nutrient-enriched vs standard formula as sole diet** MD 3.87 g/kg/d (2.26, 5.47); 5 studies/225 participants **Nutrient-enriched vs standard formula as supplement to human milk** MD 1.90 g/kg/d (0.93, 2.87); 1 study/215 participants | **Nutrient-enriched vs standard formula** MD 0.22 mm/week (-0.70, 1.13); 6 studies/440 participants  Subgroup analysis:  **Nutrient-enriched vs standard formula as sole diet** MD 1.72 mm/wk (0.23, 3.20); 4 studies/185 participants **Nutrient-enriched vs standard formula as supplement to human milk** MD -0.70 mm/wk (-1.86, 0.46); 1 study/201 participants | **Nutrient-enriched vs standard formula** MD 1.04 mm/week (0.18, 1.89); 5 studies/399 participants  Subgroup analysis:  **Nutrient-enriched vs standard formula as sole diet** MD 2.23 mm/wk (1.00, 3.52); 4 studies/185 participants **Nutrient-enriched vs standard formula as supplement to human milk** MD 0.0 mm/wk (-1.16, 1.16); 1 study/215 participants | **Nutrient-enriched vs standard formula** Skinfold thickness - triceps: MD 0.12 mm/wk (0.07, 0.17); 4 studies/364 participants Skinfold thickness - subscapular: MD 0.10 mm/wk (0.04, 0.16); 3 studies/339 participants  Subgroup analysis: **Nutrient-enriched vs standard formula as sole diet** Skinfold thickness, triceps: MD 2.26 mm/wk (1.00, 3.52); 3 studies/163 participants  Skinfold thickness, subscapular: MD 0.15 mm/wk (0.07, 0.24); 2 studies/138 participants  **Nutrient-enriched vs standard formula as supplement to human milk** Skinfold thickness, triceps: MD 0.11 mm/wk (0.05, 0.17); 1 study/201 participants  Skinfold thickness, subscapular: MD 0.15 mm/wk (0.07, 0.24); 1 study/201 participants |
|  | Young 2013 | MA | **Energy and protein-fortified vs unfortified human milk** 3-4 mo post-term: MD 138.26 g (-89.87, 366.40); 2 studies/236 participants | **Energy and protein-fortified vs unfortified human milk** 3-4 mo post term: MD 0.06 cm (-0.14, 1.33); 2 studies/236 participants | **Energy and protein-fortified vs unfortified human milk** 3-4 mo post term: MD 0.22 cm (-0.15, 0.58); 2 studies/135 participants | No outcomes reported |
|  | Young 2016 | MA | **Postdischarge formula vs standard term formula after discharge** 3-4 mo post-term: MD -7.45 g (-141.84, 126.93); 6 studies/523 participants 6 mo post-term: MD 35.54 g (-113.71, 184.78); 7 studies/576 participants  **Preterm formula vs standard term formula after discharge** 3-4 mo post-term: MD 74.41 g (-267.1, 415.93); 3 studies/130 participants 6 mo post-term: MD 74.60 g (-164.73, 313.92); 4 studies/273 participants | **Postdischarge formula vs standard term formula after discharge** 3-4 mo post-term: MD 2.45 mm (-2.01, 6.90); 6 studies/523 participants 6 mo post-term: MD 2.12 mm (-2.16, 6.41); 7 studies/576 participants  **Preterm formula vs standard term formula after discharge** 3-4 mo post-term: MD -2.27 mm (-13.09, 8.56); 3 studies/130 participants 6 mo post-term: MD 1.83 mm (-6.25, 9.92); 3 studies/160 participants | **Postdischarge formula vs standard term formula after discharge** 3-4 mo post-term: MD -0.3 mm (-2.86, 2.26); 6 studies/523 participants 6 mo post-term: MD 2.28 mm (-0.28, 4.83); 7 studies/576 participants  **Preterm formula vs standard term formula after discharge** 3-4 mo post-term: MD 3.61 mm (-2.09, 9.31); 3 studies/130 participants 6 mo post-term: MD 5.82 mm (1.32, 10.32); 3 studies/160 participants | No outcomes reported |
| Carbohydrate supplementation | | | | | | |
|  | Amissah 2018 | MA | **Carbohydrate-supplemented vs unsupplemented human milk**  Day 30: MD 160.4 g (12.4, 308.4); 1 study/75 participants | No outcomes reported | No outcomes reported | No outcomes reported |
| Fat supplementation | | | | | | |
|  | Amissah 2018 | MA | **Fat-supplemented vs unsupplemented human milk** MD 0.60 g/kg/d (-2.4, 3.6); 1 study/14 participants End of the study: MD 40.0g (−258.6, 338.6); 1 study/14 participants | **Fat-supplemented vs unsupplemented human milk** MD 0.1 cm/wk (-0.08, 0.3); 1 study/14 participants | **Fat-supplemented vs unsupplemented human milk** MD 0.2 cm/wk (-0.07, 0.40); 1 study/14 participants | No outcomes reported |
|  | Gibson 2001 | SR | **LC PUFA–supplemented formula vs standard formula** Studies reported mixed evidence regarding formula supplementation and weight gain. Three studies showed worsened weight gain in LC PUFA–supplemented infants, 7 studies showed no effect on weight gain, 1 study showed positive effect on weight gain. Trend toward worse growth outcomes when n-3 LC PUFA used in isolation, improved with combination of n-3 and n-6 LC PUFA. | **LC PUFA–supplemented formula vs standard formula**  Mixed results, but most studies showed no difference in linear growth related to LC PUFA supplementation. | **LC PUFA–supplemented formula vs standard formula** Mixed results. | **LC PUFA–supplemented formula vs standard formula Weight for length: Fish oil formula–supplemented group lower than control.** |
|  | Moon 2016 | MA | **LC PUFA–supplemented formula vs standard formula** Term: MD 0.05 kg (-0.07, 0.16); 4 studies/296 participants 2 mo post-term: MD 0.21 kg (0.08, 0.33); 5 studies/485 participants 4 mo post-term: MD 0.14 (-0.01, 0.29); 6 studies/489 participants | **LC PUFA–supplemented formula vs standard formula** Term: MD 0.34 cm (-0.27, 0.96); 4 studies/295 participants 2 mo post-term: MD 0.47 kg (0.00, 0.94); 4 studies/297 participants 4 mo post-term: MD 0.31 kg (-0.22, 0.84); 5 studies/299 participants | **LC PUFA–supplemented formula vs standard formula** Term: MD 0.18 cm (-0.26, 0.62); 3 studies/185 participants 2 mo post-term: MD 0.03 cm (-0.33, 0.38); 3 studies/187 participants 4 mo post-term: MD ‐0.09 cm (‐0.48, 0.30); 4 studies/198 participants | No outcomes reported |
|  | Nehra 2002 | MA | **High-MCT vs low-MCT formula** MD -0.35 g/kg/d (-1.44, 0.74); 5 studies/109 participants MD 2.09 g/d (-1.46, 5.64); 2 studies/42 participants  Subgroup analyses:  **31-40% MCT vs low MCT** MD 0.44 g/kg/d (-1.01, 1.89); 3 studies/62 participants **41–50% MCT vs low MCT** MD -0.86 g/kg/d (-2.45, 0.73); 2 studies/50 participants **71–80% MCT vs low MCT** MD -0.40 g/kg/d (-3.06, 2.26); 1 study/14 participants | **High-MCT vs low-MCT formula** MD 0.14 cm/wk (-0.04, 0.31); 5 studies/109 participants | **High-MCT vs low-MCT formula** MD -0.03 cm/wk (-0.15, 0.08); 5 studies/109 participants | **High-MCT vs low-MCT formula**  MD -0.15 mm/wk  (-0.41,0.11); 1 study/14 participants |
|  | Newberry 2016 | MA | **Omega-3 fatty acid–supplemented formula vs standard formula** The original review identified 20 RCTs that studied the effects of n-3 FA supplementation of preterm infants on postnatal growth patterns. Eighteen of the 20 studies found no effect on growth parameters at any time point. Two trials found that the n-3 FA–supplemented group actually had significantly lower weight at 6-18 mo than the placebo-supplemented group.  Quantitative: **4 mo with DHA+AA vs placebo**: MD -0.01 kg (-0.48, 0.47); 3 studies | **Omega-3 fatty acid– supplemented formula vs standard formula** 4 mo with DHA+AA vs placebo: MD -0.03 cm (-0.91, 0.85); 3 studies (2 from original report, 1 from update) | No outcomes reported | No outcomes reported |
|  | Rodriguez 2012 | SR | **Omega-3 LC PUFA–fortified formula vs standard formula** One study reported higher weight in infants fed with DHA and AA from algal/fungal oils vs controls and infant given single-cell algal oil DHA formula but did not note if this was significant. | **Omega-3 LC PUFA–fortified formula vs standard formula**  One study reported greater length in infants fed with DHA and AA from algal/fungal oils vs controls and infant given single-cell algal oil DHA formula but did not note if this was significant. | **Omega-3 LC PUFA–fortified formula vs standard formula**  One study found no difference in head circumference between groups. | **Omega-3 LC PUFA– fortified formula vs standard formula**  One study reported higher weight to length ratios with DHA and AA from algal/fungal oils vs controls and infant given single-cell algal oil DHA formula. Another study found lower fat-free mass in males fed DHA formula at 51 and 59 wk PMA. |
|  | Udell 2005 | MA | **ALA-enriched formula vs standard formula** (1) PMA 37-42 wk: MD -0.07 kg (-0.21, 0.06); 2 studies/96 participants (2) PMA 48 wk: MD -0.18 kg (-0.46, 0.11); 2 studies/67 participants (3) PMA 57 wk: MD -0.12 kg (-0.49, 0.26); 2 studies/58 participants | **ALA-enriched formula vs standard formula** PMA 37-42 wk: MD 0.00 cm (-0.82, 0.82); 2 studies/96 participants PMA 48 wk: MD -0.20 cm (-1.25, 0.84); 2 studies/67 participants PMA 57 wk: MD -0.09 cm (-1.40, 1.22); 2 studies/58 participants | **ALA-enriched formula vs standard formula** PMA 37-42 wk: MD -0.13 cm (-0.55, 0.29); 2 studies/96 participants PMA 48 wk: MD -0.49 cm (-1.16, 0.19); 2 studies/67 participants PMA 57 wk: MD -0.32 cm (-1.23, 0.59); 2 studies/58 participants | No outcomes reported |
| Protein supplementation | | | | | | |
|  | Amissah 2018 | MA | **Protein-supplemented vs unsupplemented human milk** MD 3.82 g/kg/d (2.94, 4.70); 5 studies/101 participants Term equivalent age: MD 61.0 g (-160.23, 282.23); 1 study/76 participants End of study: MD 250.0 g (-41.56, 541.56); 1 study/14 participants | **Protein-supplemented vs unsupplemented human milk** MD 0.12 cm/wk (0.07, 0.17) (4 studies)  Term equivalent age: MD -0.5 cm (-1.65, 0.65); 1 study/76 participants | **Protein-supplemented vs unsupplemented human milk** MD 0.06 cm/wk (0.01, 0.12); 4 studies/68 participants  Term equivalent age: MD 0.3 cm (-0.24, 0.84); 1 study/76 participants | **Protein-supplemented vs unsupplemented human milk Skinfold thickness, triceps: MD 0.06 mm/wk (-0.09, 0.21); 1 study/20 participants Skinfold thickness, subscapular: MD 0.0 mm/wk (-0.17, 0.17); 1 study/20 participants** |
|  | Cao 2018 | MA | **Taurine-supplemented formula vs standard formula** MD 0.28 g/kg/d (-0.47, 1.03); 5 subgroups from 3 studies/75 participants | **Taurine-supplemented formula vs standard formula**  MD -0.18 cm/wk (-0.27 to -0.09); 2 studies | **Taurine-supplemented formula vs standard formula**  MD 0.05 cm/wk (-0.06, t0.16); 2 studies | No outcomes reported |
|  | Fenton 2014 | MA | **High- vs low-protein formula** (1) A priori inclusion criteria: MD 2.36 g/kg/d (1.3, 3.4); 5 studies/114 participants (2) Post facto analysis: MD 2.5 g/kg/d (1.62, 3.45); 6 studies/143 participants  **Very high– vs high-protein formula** (1) A priori inclusion criteria  (a) at discharge: 3.10 g/d (-0.04, 6.24); 1 study/77 participants  (b) at term: 2.20 g/d (-1.15, 5.55); 1 study/74 participants (c) 12 wk post-term: -0.04 g/d (-0.53, 0.45); 1 study/73 participants   (2) Post facto analysis (a) MD 6.40 g/kg/d (0.38,12.42); 1 study/18 participants (b) MD 3.90 g/d (1.04,6.77); 2 studies/95 participants  **Very high– vs low-protein formula** Post facto analysis: MD -6.47 g/wk (-19.05, 6.11); 1 study/84 participants | **High- vs low-protein formula** (1) A priori inclusion criteria: MD 0.16 cm/wk (-0.02, 0.34); 2 studies/48 participants (2) Post facto analysis: MD 0.16 cm/wk (0.03, 0.30); 3 studies/77 participants  **Very high– vs high-protein formula** A priori inclusion criteria:  (a) at discharge 0.0 cm/wk (-0.14, 0.14); 1 study/77 participants  (b) at term 0.10 cm/wk (0.00,0.20);1 study/74 participants  (c) 12 wk post-term 0.0 cm/wk (-0.49, 0.49); 1 study/73 participants  **Very high– vs low- protein formula** Post facto analysis: -0.03 cm/wk (-0.10, 0.04); 1 study/84 participants | **High- vs low-protein formula** (1) A priori inclusion criteria: MD 0.37 cm/wk (0.16, 0.58); 1 study/18 participants  (2) Post facto analysis: MD 0.23 cm/wk (0.12, 0.35); 2 study/47 participants | No outcomes reported |
|  | Liu 2015 | MA | **High vs standard protein human milk fortifier** MD 1.77 g/kg/d (0.81, 2.73); 4 studies/260 participants Study end: MD 202.94 g (119.01, 286.86); 3 studies/262 participants | **High- vs standard-protein human milk fortifier** Linear growth: MD 0.21 cm/wk (0.12, 0.29); 4 studies/260 participants Study end: MD 1.12 cm (0.59, 1.64); 3 studies/262 participants | **High- vs standard-protein human milk fortifier** MD: 0.19 cm/wk (0.07, 0.31); 4 studies/260 participants Study end: MD: 0.45 cm (-0.31, 1.22); 3 studies/262 participants | No outcomes reported |
|  | Moe-Byrne 2016 | SR | **Glutamine supplementation of formula or human milk vs no glutamine supplementation** Two studies reported no statistically significant differences in the rate of weight gain or mean weight at the end of the trial period but did not provide data that could be included in meta‐analyses. One study reported higher weight gain in the glutamine group but did not report statistical significance. | **Glutamine supplementation of formula or human milk vs no glutamine supplementation** One study reported that the rate of gain in length was higher in the glutamine group but did not report numerical data. | **Glutamine supplementation of formula or human milk vs no glutamine supplementation** One study reported that the rate of gain in head circumference was higher in the glutamine group but did not report numerical data. | No outcomes reported |
|  | Pimpin 2019 | MA | **Protein-supplemented formula or human milk vs control**:  MD 0.19 kg (-0.03,0.42); 6 estimates from 5 studies/373 participants Weight-for-age Z score: MD -0.81 (-1.16,-0.46); 2 studies/169 participants | **Protein-supplemented formula or human milk vs control** MD 0.06 cm (-0.22, 0.34); 5 estimates from 4 studies/262 participants Length-for-age Z scores: MD -1.31 (-1.60, -1.01); 4 estimates from 3 studies/269 participants | Not reported | **Protein-supplemented formula or human milk vs control** Weight-for-length Z scores: MD -1.57 (-2.02,-1.12); 2 estimates from 1 study/100 participants |
|  | Tonkin 2014 | SR | **Higher- vs lower-protein supplementation of human milk or formula**  Among the 12 studies of formula with varying protein intakes, 5 studies found no difference in weight gain and 7 studies found an increase in weight parameters associated with higher protein intake. Among the 5 studies of protein-fortified vs unfortified human milk, 2 studies found no difference and 3 found significantly higher weight gain associated with protein fortification of human milk. Among the 7 studies of human milk fortifier or supplements with varying protein intake, 4 trials showed significantly increased weight gain associated with higher protein intake. | **Higher- vs lower-protein supplementation of human milk or formula** Among the studies of formula with varying protein intakes, 8 studies found no difference in attained length or rate of length gain and 1 study found an increase in length gain associated with higher protein intake. Among the studies of protein-fortified vs unfortified human milk, 3 studies found no difference in length and 2 found significantly higher length gain associated with protein fortification of human milk. Among the studies of human milk fortifier or supplements with varying protein intake, 6 trials found no difference in length and 1 found significantly increased length gain associated with higher protein intake. | **Higher- vs lower-protein supplementation of human milk or formula** Among the studies of formula with varying protein intakes, 6 studies found no difference in head circumference growth and 2 studies found an increase in head circumference growth associated with higher protein intake. Among the studies of protein-fortified vs unfortified human milk, 3 studies found no difference in length and 2 found significantly higher length gain associated with protein fortification of human milk. Among the studies of human milk fortifier or supplements with varying protein intake, 6 trials found no difference in length and 1 found significantly increased length gain associated with higher protein intake. | No outcomes reported |
| Hydrolyzation | | | | | | |
|  | Ng 2019 | MA | **Hydrolyzed vs standard formula** MD -3.02 g/kg/d (-4.55, -1.38); 3 studies/113 participants | **Hydrolyzed vs standard formula** MD -0.04 mm/wk (-1.24,1.15); 2 studies/97 participants | **Hydrolyzed vs standard formula** MD 0.27 mm/wk (-0.39,0.94); 2 studies/97 participants |  |
|  | Tan-Dy 2013 | MA | Lactase-treated vs standard formula or human milk  Day 7: MD 4.5 g/d (-0.76, 9.76); 1 study/130 participants Day 10: MD 4.9 g/d (0.18, 9.62); 1 study/130 participants Day 14: MD 2.7 g/d (-1.47, 6.87); 1 study/130 participants Study exit: MD 2.2 g/d (-0.98, 5.3); 1 study/130 participants (reported by day after study entry) | Lactase-treated vs standard formula or human milk MD 0.30 cm/wk (-0.13, 0.73); 1 study/130 participants (reported on study day 14 or study exit, whichever occurred earlier) | Lactase-treated vs standard formula or human milk  MD 0.10 cm/wk (-0.18, 0.38); 1 study/130 participants (reported on study day 14 or study exit, whichever occurred earlier) |  |

Abbreviations: AA, arachidonic acid; ALA, alpha-linolenic acid; BW, body weight; DEXA, dual-energy x-ray absorptiometry; DHA, docosahexaenoic acid; DHM, donor human milk; FA, fatty acid; LC PUFA, long-chain polyunsaturated fatty acids; MA, meta-analysis; MCT, medium-chain triglycerides; MD, mean difference; PMA, postmenstrual age; RCTs, randomized controlled trials; SR, systematic review; VLBW, very low body weight.

(9) Table A4. Strength of evidence of individual meta-analyses. Meta-analyses are grouped by quality of evidence classification, which was determined using standard umbrella review criteria. Note that P-value refers to fixed or random-effects P-value.

| **Comparison Author/Year** | **P-value** | **Heterogeneity (I^2^ value)** | **Sample size (# of cases)** | **95% confidence interval excludes the null** |
| --- | --- | --- | --- | --- |
| **Associations supported by convincing evidence** |  |  |  |  |
| None |  |  |  |  |
| **Associations supported by highly suggestive evidence** |  |  |  |  |
| None |  |  |  |  |
| **Associations supported by suggestive evidence** |  |  |  |  |
| Formula (term or preterm) vs DHM (unfortified or fortified) Linear growth (crown-heel length, mm/wk) Quigley 2019 | <0.00001 | 68 | 820 | Yes |
| Human milk fortified with both energy (carbohydrate or fat) and protein vs human milk without energy and protein fortification Length gain (cm/wk) Brown 2016 | <0.00001 | 69 | 555 | Yes |
| Human milk fortified with both energy (carbohydrate or fat) and protein vs human milk without energy and protein fortification Weight gain (g/kg/d) Brown 2016 | <0.00001 | 72 | 635 | Yes |
| Formula (term or preterm) vs DHM (unfortified or fortified) Weight gain (g/kg/d) Quigley 2019 | <0.00001 | 90 | 1028 | Yes |
| Formula (term or preterm) vs DHM (unfortified or fortified) Head growth (mm/wk) Quigley 2019 | 0.000011 | 74 | 894 | Yes |
| Human milk fortified with both energy (carbohydrate or fat) and protein vs human milk without energy and protein fortification Head growth (cm/wk) Brown 2016 | 0.000026 | 22 | 555 | Yes |
| Formula (preterm) vs DHM (fortified) Weight gain (g/kg/d) Quigley 2019 | 0.00028 | 0 | 545 | Yes |
| **Associations supported by weak evidence** |  |  |  |  |
| Protein-supplemented formula or human milk vs control Length for age Z score Pimpin 2019 | 0 | 97 | 269 | Yes |
| Protein-supplemented formula or human milk vs control Weight (kg) Pimpin 2019 | 0 | 96 | 373 | No |
| Protein-supplemented formula or human milk vs control Length (cm) Pimpin 2019 | 0 | 97 | 262 | No |
| Protein-supplemented formula or human milk vs control Weight for age Z score Pimpin 2019 | 0 | 98 | 169 | No |
| High- vs standard-protein human milk fortifier Weight achieved (g) Liu 2015 | <0.00001 | 0 | 262 | Yes |
| High- vs standard-protein human milk fortifier Linear growth (cm/wk) Liu 2015 | <0.00001 | 0 | 260 | Yes |
| High- vs standard-protein human milk fortifier Length achieved (cm) Liu 2015 | <0.00001 | 0 | 262 | yes |
| DHM vs formula Linear growth (cm/wk) Yu 2019 | <0.00001 | 0 | 307 | Yes |
| Nutrient-enriched vs standard formula as sole or supplemental diet Skinfold thickness (mm/wk) triceps Walsh 2019 | <0.00001 | 0 | 364 | No |
| Nutrient-enriched vs standard formula as sole diet Weight gain (g/kg/d) Walsh 2019 | <0.00001 | 20 | 225 | Yes |
| Formula (term or preterm) vs DHM (unfortified or fortified) Time to regain birthweight (days) Quigley 2019 | <0.00001 | 37 | 236 | Yes |
| Human milk fortified with both energy (carbohydrate or fat) and protein vs human milk without energy and protein fortification - very preterm/VLBW infants Linear growth (cm/wk) Brown 2016 | <0.00001 | 46 | 189 | Yes |
| Nutrient-enriched vs standard formula as sole or supplemental diet Weight gain (g/kg/d) Walsh 2019 | <0.00001 | 46 | 440 | Yes |
| High- vs low-protein formula, post facto Weight gain (g/kg/d) Fenton 2014 | <0.00001 | 48 | 143 | Yes |
| High- vs low-protein formula, a priori criteria Weight gain (g/kg/d) Fenton 2014 | <0.00001 | 57 | 114 | Yes |
| Formula (preterm) vs DHM (unfortified) Linear growth (crown-heel length, mm/wk) Quigley 2019 | <0.00001 | 65 | 147 | Yes |
| Formula (term or preterm) vs DHM (unfortified or fortified) as sole diet Linear growth (mm/wk) Quigley 2019 | <0.00001 | 71 | 283 | Yes |
| Protein-supplemented vs unsupplemented human milk Weight gain (g/kg/d) Amissah 2018c | <0.00001 | 73 | 101 | Yes |
| Human milk fortified with both energy (carbohydrate or fat) and protein vs human milk without energy and protein fortification, very preterm/VLBW infants Weight gain (g/kg/d) Brown 2016 | <0.00001 | 75 | 269 | Yes |
| Formula (term or preterm) vs DHM (unfortified or fortified) as sole diet Head growth (mm/wk) Quigley 2019 | <0.00001 | 77 | 305 | Yes |
| Formula (preterm) vs DHM (unfortified) Head growth (mm/wk) Quigley 2019 | <0.00001 | 84 | 221 | Yes |
| Protein-supplemented vs unsupplemented human milk Length gain (cm/wk) Amissah 2018c | <0.00001 | 89 | 68 | Yes |
| Formula (preterm) vs DHM (unfortified) Weight gain (g/kg/d) Quigley 2019 | <0.00001 | 94 | 249 | Yes |
| Formula (term or preterm) vs DHM (unfortified or fortified) as sole diet Weight gain (g/kg/d) Quigley 2019 | <0.00001 | 94 | 421 | Yes |
| Formula (term or preterm) vs DHM (unfortified or fortified) as supplemental diet Weight gain (g/kg/d) Quigley 2019 | 0.00001 | 0 | 607 | Yes |
| Formula (term) vs DHM (unfortified) Weight gain (g/kg/d) Quigley 2019 | 0.000015 | 94 | 234 | Yes |
| High- vs low-protein formula, post facto criteria Head growth (cm/wk) Fenton 2014 | 0.000083 | 59 | 47 | Yes |
| Human milk fortified with both energy (carbohydrate or fat) and protein vs human milk without energy and protein fortification in very preterm/VLBW infants Head growth (cm/wk) Brown 2016 | 0.00011 | 62 | 189 | Yes |
| High- vs standard-protein human milk fortifier Weight gain (g/kg/d) Liu 2015 | 0.00030 | 29 | 260 | Yes |
| Hydrolyzed vs standard formula Weight gain (g/kg/d) Ng 2019 | 0.00031 | 19 | 113 | Yes |
| Nutrient-enriched vs standard formula as sole diet Head growth (mm/wk) Walsh 2019 | 0.00044 | 0 | 184 | Yes |
| Nutrient-enriched vs standard formula as sole diet Skinfold thickness (mm/wk) subscapular Walsh 2019 | 0.00061 | 0 | 138 | Yes |
| Nutrient-enriched vs standard formula as sole or supplemental diet Skinfold thickness (mm/wk) subscapular Walsh 2019 | 0.00067 | 25 | 339 | Yes |
| Very high– vs high-protein formula, post facto Weight gain (g/d & g/kg/d) Fenton 2014 | 0.00094 | 4 | 113 | Yes |
| LC PUFA–supplemented formula vs standard formula Weight at 2 mo post-term (kg) Moon 2016 | 0.00099 | 69 | 485 | Yes |
| Taurine-supplemented formula vs standard formula  Length gain (cm/wk) Cao 2018 | <0.001 | 0 | <216 | Yes |
| Human milk fortified with both energy (carbohydrate or fat) and protein vs human milk without energy and protein fortification, low- and middle-income countries Weight gain (g/kg/d) Brown 2016 | 0.0016 | 48 | 214 | Yes |
| High vs standard protein human milk fortifier Head growth (cm/wk) Liu 2015 | 0.002 | 56 | 260 | Yes |
| Formula (preterm) vs DHM (fortified) Linear growth (crown-heel length, mm/wk) Quigley 2019 | 0.0049 | 83 | 545 | Yes |
| DHM vs formula Weight gain (g/d) Yu 2019 | 0.005 | 92 | 231 | No |
| Nutrient-enriched vs standard formula as sole diet Skinfold thickness (mm/wk) triceps Walsh 2019 | 0.0051 | 0 | 163 | Yes |
| Preterm formula vs term formula, 6 mo post-term Head circumference (mm) Young 2016 | 0.011 | 24 | 160 | Yes |
| High- vs low-protein formula, post facto Linear growth (cm/wk) Fenton 2014 | 0.015 | 0 | 77 | Yes |
| Formula (term) vs DHM (unfortified) Head growth (mm/wk) Quigley 2019 | 0.016 | 0 | 128 | Yes |
| Nutrient-enriched vs standard formula as sole or supplemental diet Head growth (mm/wk) Walsh 2019 | 0.017 | 57 | 399 | Yes |
| DHM vs formula Head growth (cm/wk) Yu 2019 | 0.02 | 47 | 206 | No |
| Protein-supplemented vs unsupplemented human milk Head growth (cm/wk) Amissah 2018c | 0.024 | 84 | 68 | Yes |
| Formula (term) vs DHM (unfortified) Linear growth (crown-heel length, mm/wk) Quigley 2019 | 0.024 | 0 | 128 | Yes |
| Nutrient-enriched vs standard formula as sole diet Linear growth (mm/wk) Walsh 2019 | 0.024 | 47 | 185 | Yes |
| LC PUFA–supplemented formula vs standard formula Length at 2 mo post-term (cm) Moon 2016 | 0.048 | 0 | 297 | No |
| **Nonsignificant associations** |  |  |  |  |
| Formula (term or preterm) vs DHM (unfortified or fortified) as supplemental diet Linear growth (mm/wk) Quigley 2019 | 0.065 | 56 | 537 | No |
| LC PUFA–supplemented formula vs standard formula Weight at 4 mo post-term (kg) Moon 2016 | 0.072 | 55 | 489 | No |
| High vs low protein, a priori Linear growth (cm/wk) Fenton 2014 | 0.081 | 42 | 48 | No |
| Postdischarge formula vs term formula at 6 mo post-term Head circumference (mm) Young 2016 | 0.081 | 69 | 576 | No |
| Energy and protein-fortified vs unfortified human milk Length (cm) at 3-4 mo corrected age Young 2013 | 0.11 | 72 | 236 | No |
| High vs low MCT formula Linear growth (cm/wk) Nehra 2002 | 0.13 | 50 | 109 | No |
| Preterm formula vs term formula at 3-4 mo post-term Head circumference (mm) Young 2016 | 0.21 | 0 | 130 | No |
| Energy and protein-fortified vs unfortified human milk Weight (g) at 3-4 mo corrected age Young 2013 | 0.23 | 40 | 236 | No |
| High- vs standard-protein human milk fortifier Head circumference achieved (cm) Liu 2015 | 0.24 | 67 | 262 | No |
| Energy and protein-fortified vs unfortified human milk Head circumference (cm) at 3-4 mo corrected age Young 2013 | 0.24 | 84 | 235 | No |
| High vs low MCT formula Weight gain (g/d) Nehra 2002 | 0.25 | 0 | 42 | No |
| LC PUFA–supplemented formula vs standard formula Length at 4 mo post-term (cm) Moon 2016 | 0.25 | 26 | 299 | No |
| LC PUFA–supplemented formula vs standard formula Length at term (cm) Moon 2016 | 0.27 | 59 | 295 | No |
| Postdischarge formula vs term formula at 3 to 4 mo post-term Length (crown-heel, mm) Young 2016 | 0.28 | 81 | 523 | No |
| 41–50 MCT vs low MCT formula Weight gain (g/kg/d) Nehra 2002 | 0.29 | 0 | 50 | No |
| Formula (preterm) vs DHM (fortified) Head growth (mm/wk) Quigley 2019 | 0.3 | 0 | 545 | No |
| Postdischarge formula vs term formula at 6 mos post-term Length (crown-heel, mm) Young 2016 | 0.33 | 75 | 576 | No |
| Nutrient-enriched vs standard formula Time to regain birthweight (days) Walsh 2019 | 0.37 | 57 | 74 | No |
| Taurine-supplemented formula vs standard formula  Head growth (cm/wk) Cao 2018 | 0.377 | 42 | <216 | No |
| Formula (term or preterm) vs DHM (unfortified or fortified) as supplemental diet Head growth (mm/wk) Quigley 2019 | 0.39 | 0 | 589 | No |
| LC PUFA–supplemented formula vs standard formula Head circumference at term (cm) Moon 2016 | 0.42 | 31 | 185 | No |
| LC PUFA-supplemented formula vs standard formula Weight at term (kg) Moon 2016 | 0.42 | 46 | 296 | No |
| Hydrolyzed vs standard formula Head growth (mm/wk) Ng 2019 | 0.42 | 82 | 97 | No |
| Taurine-supplemented formula vs standard formula  Weight gain (g/kg/d) Cao 2018 | 0.46 | 57 | 75 | No |
| High vs low MCT formula Weight gain (g/kg/d) Nehra 2002 | 0.53 | 0 | 109 | No |
| Preterm formula vs term formula at 6 mo post-term Weight (g) Young 2016 | 0.54 | 0 | 273 | No |
| 31–40 MCT vs low MCT formula Weight gain (g/kg/d) Nehra 2002 | 0.55 | 0 | 62 | No |
| High vs low MCT formula Head growth (cm/wk) Nehra 2002 | 0.56 | 0 | 109 | No |
| Post discharge formula vs term formula at 6 mo post-term Weight (g) Young 2016 | 0.64 | 64 | 576 | No |
| Nutrient-enriched vs standard formula as sole or supplemental diet Linear growth (mm/wk) Walsh 2019 | 0.64 | 67 | 386 | No |
| Preterm formula vs term formula at 6 mo post-term  Length (crown-heel, mm) Young 2016 | 0.66 | 0 | 160 | No |
| LC PUFA–supplemented formula vs standard formula Head circumference at 4 mo post-term (cm) Moon 2016 | 0.66 | 54 | 198 | No |
| Preterm formula vs term formula at 3 to 4 mo post-term Weight (g) Young 2016 | 0.67 | 46 | 130 | No |
| Preterm formula vs term formula at 3 to 4 mo post-term Length (crown-heel, mm) Young 2016 | 0.68 | 40 | 130 | No |
| Protein-supplemented formula or human milk vs control Weight-for-length Z score Pimpin 2019 | 0.71 | 0 | 100 | Yes |
| Postdischarge formula vs term formula at 3 to 4 mo post-term Head circumference (mm) Young 2016 | 0.82 | 71 | 523 | No |
| LC PUFA–supplemented formula vs standard formula Head circumference at 2 mo post-term (cm) Moon 2016 | 0.89 | 41 | 187 | No |
| Postdischarge formula vs term formula at 3 to 4 mo post-term Weight (g) Young 2016 | 0.91 | 62 | 523 | No |
| Hydrolyzed vs standard formula Linear growth (mm/wk) Ng 2019 | 0.94 | 0 | 97 | No |
| **Associations not adequately assessed due to absence of data** |  |  |  |  |
| Omega-3 fatty acid supplementation vs standard formula Weight at 4 mo (kg), DHA+AA vs placebo Newberry 2016 |  |  |  |  |
| Omega-3 fatty acid supplementation vs standard formula Length at 4 mo (cm), DHA+AA vs placebo Newberry 2016 |  |  |  |  |
| ALA-enriched formula vs standard formula Weight: (1) PMA 37–42 wk (2) PMA 48 wk (3) PMA 57 wk Udell 2005 |  |  |  |  |
| ALA-enriched formula vs standard formula Length: (1) PMA 37–42 wk (2) PMA 48 wk (3) PMA 57 wk Udell 2005 |  |  |  |  |

Abbreviations: AA, arachidonic acid; ALA, alpha-linolenic acid; DHA, docosahexaenoic acid; DHM, donor human milk; LC PUFA, long-chain polyunsaturated fatty acids; PMA, postmenstrual age; VLBW, very low birthweight.
